# Design, Identification, and Evolution of a Surface Ruthenium(II/III) Single Site for CO Activation

**DOI:** 10.1002/anie.202008370

**Published:** 2020-11-13

**Authors:** Liqun Kang, Bolun Wang, Adam Thetford, Ke Wu, Mohsen Danaie, Qian He, Emma K. Gibson, Ling‐Dong Sun, Hiroyuki Asakura, C. Richard A. Catlow, Feng Ryan Wang

**Affiliations:** ^1^ Department of Chemical Engineering University College London London WC1E 7JE UK; ^2^ Department of Chemistry The University of Manchester Manchester M13 9PL UK; ^3^ College of Chemistry and Molecular Engineering Peking University Beijing 100871 China; ^4^ Electron Physical Science Imaging Centre Diamond Light Source Didcot OX11 0DE UK; ^5^ Department of Materials Science and Engineering National University of Singapore Singapore 117575 Singapore; ^6^ School of Chemistry University of Glasgow Glasgow G12 8QQ UK; ^7^ Elements Strategy Initiative for Catalysts & Batteries Kyoto University Kyoto 615-8245 Japan; ^8^ Department of Chemistry University College London London WC1H 0AJ UK; ^9^ School of Chemistry Cardiff University Cardiff CF10 3AT UK

**Keywords:** heterogeneous catalysis, ruthenium(II/III) complexes, single site, surface coordination chemistry, X-ray absorption spectroscopy

## Abstract

Ru^II^ compounds are widely used in catalysis, photocatalysis, and medical applications. They are usually obtained in a reductive environment as molecular O_2_ can oxidize Ru^II^ to Ru^III^ and Ru^IV^. Here we report the design, identification and evolution of an air‐stable surface [bipy‐Ru^II^(CO)_2_Cl_2_] site that is covalently mounted onto a polyphenylene framework. Such a Ru^II^ site was obtained by reduction of [bipy‐Ru^III^Cl_4_]^−^ with simultaneous ligand exchange from Cl^−^ to CO. This structural evolution was witnessed by a combination of in situ X‐ray and infrared spectroscopy studies. The [bipy‐Ru^II^(CO)_2_Cl_2_] site enables oxidation of CO with a turnover frequency of 0.73×10^−2^ s^−1^ at 462 K, while the Ru^III^ site is completely inert. This work contributes to the study of structure–activity relationship by demonstrating a practical control over both geometric and electronic structures of single‐site catalysts at molecular level.

## Graphical abstract

An air‐stable surface [bipy‐Ru^II^(CO)_2_Cl_2_] single site is designed towards CO oxidation, while all other Ru^III^ single sites are not active. The methodology is further extended to nine transition‐metal single‐site systems, enabling the use of surface coordination chemistry in heterogeneous catalysis.

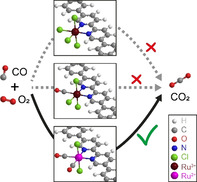

## Introduction

Ru^II^ complexes were first discovered in the 19th century.^[1]^ Their chemistry is diverse and has sparked wide interest in homogeneous catalysis,^[2]^ photocatalysis,^[3]^ and biochemical and biomedical applications.^[4]^ Most Ru^II^ compounds function as reducing agents and only a few Ru^II^ complexes with strong‐field π‐acceptor ligands are air‐stable.[^1^, ^5^] The standard reduction potentials *E* ^⊖^ of Ru^III^/Ru^II^ in [Ru(H_2_O)_6_]^3+^/ [Ru(H_2_O)_6_]^2+^, [Ru(NH_3_)_6_]^3+^/ [Ru(NH_3_)_6_]^2+^, [Ru(en)_6_]^3+^/ [Ru(en)_6_]^2+^, [Ru(CN)_6_]^3−^/ [Ru(CN)_6_]^4−^ and [Ru(bipy)_6_]^3+^/ [Ru(bipy)_6_]^2+^ pairs are 0.23 V, 0.10 V, 0.21 V, 0.86 V and 1.24 V, respectively.^[6]^ Nonetheless, the Ru^II^ system can be oxidized by molecular O_2_ at elevated temperatures, limiting its study and application in heterogeneous oxidation chemistry.

Inspired by the relatively high reduction potential of [Ru(bipy)_6_]^3+^/[Ru(bipy)_6_]^2+^ pair, we covalently bind bidentate bipyridine (bipy) ligand into the polyphenylene (PPhen) framework via a C−C coupling reaction, converting the bipy ligands from solute molecules into surface binding sites. This enables the design of surface single‐site and single‐atom catalysts, which mimic molecular catalysts and have unique coordination and electron configuration compared with particles and clusters.^[7]^ These reported single sites, usually supported by metal oxide or carbon, have shown unique activity or selectivity in the water‐gas‐shift reaction,^[8]^ hydrochlorination,^[9]^ methane activation,^[10]^ CH_3_OH steam reforming,^[11]^ and hydrogenation.^[12]^ In this work, we successfully designed and prepared the surface [bipy‐Ru^II^(CO)_2_Cl_2_] single site via simultaneous Ru^III^ to Ru^II^ transition and Cl^−^/CO ligand exchange. Such solid/gas interface coordination chemistry, including both geometric and electronic structures, is monitored by a combination of in situ X‐ray absorption fine structure (XAFS), diffuse reflectance infrared Fourier transform spectroscopy (DRIFTS), ex situ far‐FTIR, X‐ray photoelectron spectroscopy (XPS), and density functional theory (DFT) calculations. The [bipy‐Ru^II^(CO)_2_Cl_2_] site is stable in air for at least 400 days. It enables CO oxidation with a turnover frequency (TOF) of 0.73×10^−2^ s^−1^ at 462 K, while the Ru^III^ site is completely inert. Such distinct catalytic performance could result from the geometric configuration of the [bipy‐Ru^II^(CO)_2_Cl_2_] site and its optimized energy level that boost CO conversion.

Building a surface single site on PPhen with bipy ligands is proven to be a general and versatile method. Eleven types of uniform and well‐defined surface single sites are obtained, including [bipy‐FeCl_4_]^−^, [bipy‐CoCl_2_], [bipy‐Ni(H_2_O)_4_]^2+^, [bipy‐CuCl_2_], [bipy‐Cu(H_2_O)_4_]^2+^, [bipy‐ZnCl_2_], [bipy‐RuCl_4_]^−^, [bipy‐RuBr_4_]^−^, [bipy‐RhCl_4_]^−^, [bipy‐PdCl_2_], and [bipy‐PtCl_4_]. This variety of selection including both metal cations and ligands suggests great potential in catalysis and mechanistic understandings.

## Results and Discussion

### Design and determination of the [bipy‐Ru^III^Cl_4_]^−^ single site.

**Figure 1:**
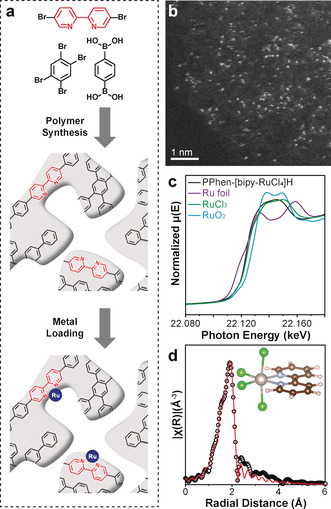
Identification of [bipy‐Ru^III^Cl_4_]^−^ single site. a) The general approach in creating the single‐site coordination environment. b) HAADF‐STEM images of PPhen‐[bipy‐Ru^III^Cl_4_]H. c) Ru K‐edge XANES spectra of PPhen‐[bipy‐Ru^III^Cl_4_]H, Ru foil, RuCl_3_, and RuO_2_. d) Experimental and fitted EXAFS data of PPhen‐[bipy‐Ru^III^Cl_4_]H. The inlet is the DFT simulation of the [bipy‐Ru^III^Cl_4_]^−^ structure.

Our strategy utilizes Suzuki cross‐coupling with 5,5′‐dibromo‐2,2′‐bipyridine, 1,2,4,5‐tetrabromobenzene, and benzene‐1,4‐diboronic acid as the building blocks (Figure 1 a). The latter two comprise the backbone of the PPhen framework^[13]^ while 5,5′‐dibromo‐2,2′‐bipyridine provides bipy ligand functionality. We choose PPhen as the backbone for its pure aromatic structure, microporosity and high thermal stability under air (up to 400 °C), as reported previously.^[13]^ Bipy is a typical chelating ligand that can stabilize metal cations.^[14]^ It has been functionalized within the UiO‐67^[15]^ and polystyrene system recently.^[16]^ The ratio between 5,5′‐dibromo‐2,2′‐bipyridine and 1,2,4,5‐tetrabromobenzene determines the amount of bipy ligands in relation to the PPhen‐bipy polymer, thereby the loading of single metal sites. The ratio is set to 1:3 for that raising the content of dibromo molecule will increase the linear content of the polymer, decrease the overall surface area and consequently reduce the accessibility to the bipy sites.^[17]^ The PPhen‐bipy is Pd free, as shown in the energy dispersive spectroscopy (EDS; Supporting Information, Figure S1).

The PPhen‐bipy reacts with RuCl_3_ to form a [bipy‐RuCl_4_]^−^ single site in the presence of HCl, in which the bipy ligand anchors the Ru^3+^ while Cl^−^ anions are balancing the +3 charge. High density and good dispersion of single sites are achieved, with more than 200 Ru ions identified over 40 nm^2^ of high angle annular dark field‐scanning transmission electron microscopy (HAADF‐STEM) image (Figure 1 b). The loading of Ru and Cl is confirmed in the EDS and X‐ray photoelectron spectroscopy (Supporting Information, Figures S2, S3). The absorption edge position of Ru K‐edge X‐ray absorption near‐edge structure (XANES) is almost identical to RuCl_3_ rather than Ru^0^ foil or RuO_2_, suggesting that the oxidation state is Ru^III^ (Figure 1 c). The extended X‐ray absorption fine structure (EXAFS) further confirms the single‐site nature, showing only Ru−N and Ru−Cl scattering with no Ru−Ru scattering (Figure 1 d). The coordination numbers and bond lengths of Ru−N and Ru−Cl scattering are 2.0 at 2.05±0.02 Å and 4.0±0.4 at 2.35±0.01 Å, respectively (Supporting Information, Table S1). The calculated bipy‐Ru^III^Cl_4_^−^ structure using DFT (Figure 1 d inlet; Supporting Information, Figure S4a) shows very similar values of Ru−N at 2.096 Å and Ru−Cl at 2.400 Å (Supporting Information, Table S2). The bond lengths in both EXAFS and DFT calculations match the reported value of molecular K[bipy‐Ru^III^Cl_4_] complex (2.36 Å for Ru−Cl and 2.02 Å for Ru−N; Supporting Information, Table S3 entry 18).^[18]^ The PPhen‐[bipy‐Ru^III^Cl_4_]^−^ is stable in air up to 350 °C (Supporting Information, Figure S5), suggesting its capability for reactions at elevated temperature even in oxidative environments. The 5.8 wt % Ru loading is calculated from the difference in weight residue between the PPhen‐[bipy‐Ru^III^Cl_4_]^−^ and PPhen‐bipy. The value is smaller than the circa 8.6 wt % Ru loading calculated from the ratio of the bipy ligand in the PPhen‐bipy (see calculation in Supporting Information). The possible reasons are the loss of bipy during PPhen‐bipy synthesis and the formation of inaccessible bipy site. The BET equivalent surface area is 465 m^2^ g^−1^, providing the porous framework for heterogeneous catalysis (Supporting Information, Figure S6 and Table S4). Obtaining such well‐defined [bipy‐Ru^III^Cl_4_]^−^ site is crucial for determining the solid/gas interface coordination chemistry, which provides the possibility to trace further structure evolution.

### Structural Evolution from [bipy‐Ru^III^Cl_4_]^−^ to mer(Cl)‐[bipy‐Ru^III^(CO)Cl_3_]

**Figure 2:**
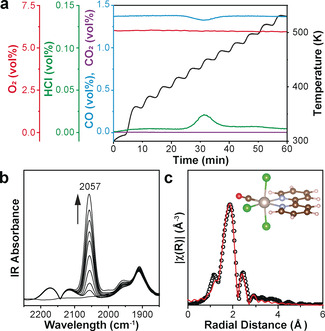
Evolution from [bipy‐Ru^III^Cl_4_]^−^ to *mer*(Cl)‐[bipy‐Ru^III^(CO)Cl_3_]. a) Mass spectrometry (MS) showing the outlet gas profile with the corresponding temperature profile under 1 %CO and 5 %O_2_ flow, O_2_ is fed to monitor if there is any oxidation of CO to CO_2_. Blue CO, purple CO_2_, red O_2_, green HCl. b) In situ DRIFTS spectra, showing the formation of one terminal CO coordination at 2057 cm^−1^. c) Experimental and fitted EXAFS data, suggesting the [bipy‐Ru^III^(CO)Cl_3_] single‐site structures with corresponding DFT calculated models.

H_2_ reduces [bipy‐Ru^III^Cl_4_]^−^ into Ru nanoparticles (Supporting Information, Figure S7), owing to the fact that H_2_ takes away Cl‐ and no other ligand is available at the surface to maintain the hexacoordinated octahedral structure. Consequently, CO, as a reductive gas phase ligand, is selected for this ligand exchange reaction at the solid/gas interface. O_2_ is co‐fed, thereby any detection of the Ru^II^ will guarantee its stability under molecular O_2_. At room temperature, the initial [bipy‐Ru^III^Cl_4_]^−^ remains unchanged (Supporting Information, Figure S8), showing no ligand exchange with CO. The release of HCl is observed starting from 433 K (Figure 2 a). A surface adsorbed C≡O vibration is found in the DRIFTS spectra at 2057 cm^−1^ (Figure 2 b). This vibration absorption corresponds well to the FTIR spectrum of molecular *mer*(Cl)‐[Ru(bipy)(CO)Cl_3_] (Supporting Information, Table S5 complex D).^[19]^ The fitted EXAFS spectrum shows the decrease of Ru−Cl coordination number from 4.0±0.4 to 3.2±0.2 while maintaining the bond distance at 2.35±0.01 Å (Figure 2 c; Supporting Information, Figures S9b, S10b, Table S1). A new Ru‐CO scattering is observed at 1.88±0.02 Å with a coordination number of 1.1±0.2 (Figure 2 c; Supporting Information, Table S1). The bond lengths are the same as the values reported for molecular *mer*(Cl)‐[Ru(bipy)(CO)Cl_3_] (Ru−N 2.08 Å, Ru−Cl 2.33 Å, and Ru−C 1.89 Å; Supporting Information, Table S5 complex D).^[19]^ Similar bond lengths are also confirmed by the DFT calculations (Supporting Information, Figure S4b, Table S2). No obvious edge shift is observed in the XANES, suggesting the unchanged oxidation state of Ru^III^ (Supporting Information, Figure S9a, S10a). We conclude that majority of the Ru^III^ surface complex becomes *mer*(Cl)‐[bipy‐Ru^III^(CO)Cl_3_] via:

(1) $$\left[\mathrm{bipy}\text{-}\mathrm{Ru}{}^{\mathrm{III}}\mathrm{Cl}{}_{4}\right]H+\mathrm{CO}\to mer\left(\mathrm{Cl}\right)\text{-}\left[\mathrm{bipy}\text{-}\mathrm{Ru}{}^{\mathrm{III}}\left(\mathrm{CO}\right)\mathrm{Cl}{}_{3}\right]+\mathrm{HCl}$$

*mer*(Cl)‐[bipy‐Ru^III^(CO)Cl_3_] is relatively stable because any removal of the Cl^−^ will result in the formation of unfavorable positively charged Ru complexes. With 100 mg of PPhen‐[bipy‐Ru^III^Cl_4_]H, 1.25 mL of CO is consumed, corresponding to 5.6 wt % Ru loading on the initial PPhen‐[bipy‐Ru^III^Cl_4_]H (Supporting Information, Figure S11). This observation is in good agreement with the 5.8 wt % Ru loading calculated from the Thermogravimetric analysis (TGA), proving the chemical stoichiometry of Equation (1).

### Structural Evolution from mer(Cl)‐[bipy‐Ru^III^(CO)Cl_3_] to cis(CO)‐trans(Cl)‐[bipy‐Ru^II^(CO)_2_Cl_2_]

**Figure 3:**
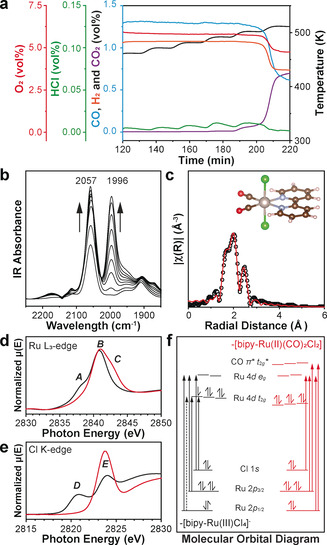
Structural evolution from *mer*(Cl)‐[bipy‐Ru^III^(CO)Cl_3_] to *cis*(CO)‐*trans*(Cl)‐[bipy‐Ru^II^(CO)_2_Cl_2_]. a) Mass spectrometry showing the outlet gas profile with corresponding temperature profile: Blue CO, purple CO_2_, red O_2_, green HCl, orange H_2_. b) In situ DRIFTS spectra showing the formation of two terminal CO coordination at 2057 cm^−1^ and 1996 cm^−1^. c) Experimental and fitted EXAFS results, suggesting the [bipy‐Ru^II^(CO)_2_Cl_2_] single‐site structures with corresponding DFT calculated models. XANES of [bipy‐Ru^III^Cl_4_]^−^ and *cis*(CO)‐*trans*(Cl)‐[bipy‐Ru^II^(CO)_2_Cl_2_] at d) Ru L_3_‐edge and e) Cl K‐edge. f) Molecular orbital diagram of Ru^III^ and Ru^II^ single sites.

A further increase in the temperature does not result in any significant changes in the online MS profile, in situ DRIFTS and XAFS spectra (Figure 2 a; Supporting Information, Figure S9). This shows that CO can only replace Cl^−^, while the reduction of Ru^3+^ requires H_2_. H_2_ is added to the feed in order to reduce Ru and remove another Cl^−^ ligand. HCl is detected in the MS, suggesting the reaction between H_2_ and the Cl^−^ ligand (Figure 3 a). DRIFTS and FTIR spectra show a second C≡O stretching vibration absorption at 1996 cm^−1^ when H_2_ is introduced (Figure 3 b; Supporting Information, Figure S12a). The far‐FTIR spectra shows only one type of Ru−Cl stretching at 331 cm^−1^ (Supporting Information, Figure S12b). These results match the FTIR spectrum of molecular *cis*(CO)‐*trans*(Cl)‐[Ru^II^(bipy)(CO)_2_Cl_2_] (Table S5, complex E)^[20]^ but are different from *cis*(CO)‐*cis*(Cl)‐[Ru^II^(bipy)(CO)_2_Cl_2_] (Supporting Information, Table S5, complex F, 2040 and 1980 cm^−1^).^[20]^ From analysis of the EXAFS spectrum, the Ru−Cl coordination number is reduced to 2.2±0.3 with a slight increase of bond length to 2.39±0.01 Å, confirming the ligand exchange of Cl^−^ via CO with the help of H_2_ (Figure 3 c; Supporting Information, Figure S10d and Table S1). As a result, the coordination number of Ru−CO at 1.88±0.01 Å increases to 1.9±0.3 (Supporting Information, Table S1).^[21]^ DFT calculations are performed accordingly with *cis*(CO)‐*trans*(Cl)‐[Ru(bipy)(CO)_2_Cl_2_], showing similar Ru−Cl bond length (2.339 Å) and a slightly longer Ru−CO bond length (2.002 Å; Supporting Information, Figure S4c, Table S2).

Ru L‐edge and Cl K‐edge XAFS spectra were measured to determine the oxidation state of Ru. At Ru L_3_‐edge region, two absorption features *A* at 2838.0 eV and *B* at 2840.9 eV have been found for the initial [bipy‐Ru^III^Cl_4_]^−^ site (Figure 3 d; Supporting Information, Figure S13 and Table S6). According to previous research and in accordance with the dipole selection rule,^[22]^ these two peaks are attributed to 2p_3/2_→4d t_2g_ and 2p_3/2_→4d e_g_ transitions (Figure 3 f), respectively.^[23]^ This proves that Ru 4d t_2g_ orbitals are not completely filled and are in the form of Ru^III^ t_2g_^5^e_g_^0^, which is in good agreement with the Ru K‐edge XANES spectrum (Figure 1 c). For *cis*(CO)‐*trans*(Cl)‐[Ru(bipy)(CO)_2_Cl_2_] sites, feature *A* disappears, and a new feature *C* appears at 2843.0 eV. The absence of feature *A* suggests completely filled Ru^II^ 4d t_2g_^6^ orbitals. Thus, the reduction from Ru^III^ to Ru^II^ is confirmed. The additional feature *C* is assigned to Ru 2p_3/2_→CO π* transition.[^22^, ^24^] The CO π* orbitals are formed above Ru 4d e_g_ as a result of Ru−CO π backbonding. Similar electronic structures are also obtained from the Ru L_2_‐edge XAFS spectra (Supporting Information, Figures S13, S14). The Ru K‐edge spectra show no obvious changes, resulting from the dipole forbidden 1s→4d transition that prevents the detection of valence orbitals (Supporting Information, Figures S10c, S15, Table S7).

In Ru‐Cl_*x*_ complexes, d–p hybridization enables the transition from Cl 1s to Ru 4d,^[25]^ thereby Cl K‐edge XAFS can also validate the Ru oxidation state. There is a strong pre‐edge absorption feature *E* at 2823.8 eV for both [bipy‐Ru^III^Cl_4_]^−^ and *cis*(CO)‐*trans*(Cl)‐[Ru^II^(bipy)(CO)_2_Cl_2_] sites in the Cl K‐edge XAFS, representing the Cl 1s→Ru 4d e_g_ transition (Figure 3 e). The [bipy‐Ru^III^Cl_4_]^−^ site shows a feature *D* at 2820.8 eV, which is absent in *cis*(CO)‐*trans*(Cl)‐[Ru^II^(bipy)(CO)_2_Cl_2_] site. This feature originates from Cl 1s to unfulfilled Ru^III^ 4d t_2g_. Therefore, the reduction is further verified via Cl K‐edge XAFS. It also suggests that the Cl is not an outer shell counter anion but is in a close coordination environment with Ru cation.[^25b^, ^26^] The energy differences between features *A* and *B* as well as *D* and *E* are 2.9 eV and 3.0 eV, respectively. Such ligand field splitting energy Δ_0_ at core hole state is between 23 400 to 24 200 cm^−1^, which corresponds well with the absorption at 352 nm in UV/Vis spectra (Supporting Information, Figure S16). Furthermore, XPS at the Ru 3d region also confirms the change of oxidation states, showing a decrease of the Ru 3d_5/2_ binding energy from 282.4 eV to 281.8 eV (Supporting Information, Figure S17 and Table S8). As such, we confirm that the reaction mechanism followed is:

(2) $$2\;mer\left(\mathrm{Cl}\right)\text{-}\left[\mathrm{bipy}\text{-}\mathrm{Ru}{}^{\mathrm{III}}\left(\mathrm{CO}\right)\mathrm{Cl}{}_{3}\right]+2\;\mathrm{CO}+H{}_{2}\to 2\;cis\left(\mathrm{CO}\right)\text{-}trans\left(\mathrm{Cl}\right)\text{-}[\mathrm{Ru}{}^{\mathrm{II}}\left(\mathrm{bipy}\right)\left(\mathrm{CO}\right){}_{2}\mathrm{Cl}{}_{2}]+2\;\mathrm{HCl}$$

Molecular O_2_ is constantly present in the system and an air stable *cis*(CO)‐*trans*(Cl)‐[Ru^II^(bipy)(CO)_2_Cl_2_] site is achieved.

### CO Oxidation over Ru^II^ and Ru^III^ Single Sites

Further increasing the temperature shows the release of CO_2_ from 503 K (Figure 3 a). This suggests that *cis*(CO)‐*trans*(Cl)‐[Ru^II^(bipy)(CO)_2_Cl_2_] is active for CO oxidation. We perform CO oxidation for both *mer*(Cl)‐[bipy‐Ru^III^(CO)Cl_3_] and *cis*(CO)‐*trans*(Cl)‐[Ru^II^(bipy)(CO)_2_Cl_2_] sites. The former shows no conversion, whereas the latter gives a light off at 388 K and 35 % conversion at 453 K, *p*_CO_=0.01 bar and weight hourly space velocity (WHSV) of 60,000 mL h^−1^ g^−1^ (Supporting Information, Figure S18). An activation energy (*E*_a_) of 90 kJ mol^−1^ is determined at the WHSV of 600 000 mL h^−1^ g^−1^. Based on the EXAFS study of the *cis*(CO)‐*trans*(Cl)‐[Ru^II^(bipy)(CO)_2_Cl_2_] site and its narrow full width at half maximum (FWHM) of CO absorption in FTIR spectra (Supporting Information, Figure S12a), we assume that all the Ru^II^ sites are identical and perform the same in CO oxidation. The corresponding TOFs are then calculated as 0.73×10^−2^ s^−1^, 1.77×10^−2^ s^−1^, and 3.89×10^−2^ s^−1^ at 462, 479 and 498 K, respectively. The TOF and *E*_a_ are similar to the state‐of‐the‐art Cu and Pt single site over metal oxide support for CO oxidation (Supporting Information, Table S9). HAADF‐STEM images show the high density of Ru^2+^ cations well dispersed on the polymer framework after catalysis for *cis*(CO)‐*trans*(Cl)‐[Ru^II^(bipy)(CO)_2_Cl_2_] site (Supporting Information, Figure S19). There is no sign of aggregation or cluster formation, which is further proved via ex situ XRD studies (Supporting Information, Figure S19a–f). The Ru^II^ retains the *cis*(CO)‐*trans*(Cl)‐[Ru^II^(bipy)(CO)_2_Cl_2_] structure, as indicated in the XAFS (Supporting Information, Figure S19g–i) and FTIR (Supporting Information, Figure S12c) studies after catalysis. The PPhen‐bipy polymer remains unchanged, according to the post catalysis FTIR and scanning electron microscopy analysis (Supporting Information, Figure S12c and Figure S20). This is because of its high stability in air at elevated temperatures (Supporting Information, Figure S5c). The activity of *cis*(CO)‐*trans*(Cl)‐[Ru^II^(bipy)(CO)_2_Cl_2_] is stable during the 24 h on stream stability test (Supporting Information, Figure S21). The distinct differences in CO oxidation activity between *mer*(Cl)‐[bipy‐Ru^III^(CO)Cl_3_] single site and *cis*(CO)‐*trans*(Cl)‐[Ru^II^(bipy)(CO)_2_Cl_2_] single site are resulted from their geometric and electronic properties. Geometrically, the former has three Cl^−^ ligands, which are strong binding ligands in metal complexes (poisonous for metal active center) and leaves only one CO ligand to be exchanged during catalysis. In comparison, the latter offers two exchangeable CO ligands in *cis* configuration, enabling the local activation of CO within the Ru coordination environment. Electronically, the Ru^II^ is in a reduced form, which is reported to activate CO in water‐gas‐shift reaction.^[27]^

The role of the halogen ligand is further studied via [bipy‐Ru^III^Br_4_]^−^ single site, where Br^−^ is a better leaving group than Cl^−^ according to solution based coordination chemistry. The transition from [bipy‐Ru^III^Br_4_]^−^ to [bipy‐Ru^III^(CO)Br_3_] takes place at room temperature while the reduction from [bipy‐Ru^III^(CO)Br_3_] to [bipy‐Ru^II^(CO)_2_Br_2_] requires 513 K (Supporting Information, Figure S22 and Table S2). This is 40 K higher than the temperature required to reduce the Cl coordinated Ru single sites. Without the presence of solvent, the bond breaking and formation here at the solid/gas interface are different.

### General Synthetic Strategy

**Figure 4:**
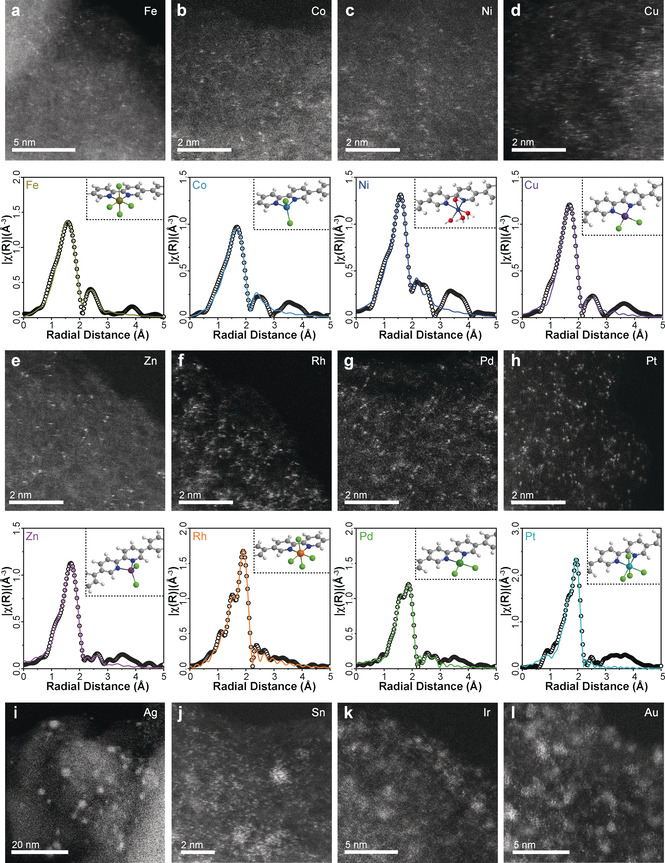
HAADF‐STEM and EXAFS identifications of single site PPhen‐[bipy‐MX_n_] structure. Cs‐corrected HAADF‐STEM images show the single metal ions supported on PPhen‐bipy for a) Fe^3+^, b) Co^2+^, c) Ni^2+^, d) Cu^2+^, e) Zn^2+^, f) Rh^3+^, g) Pd^2+^, and h) Pt^4+^. Clusters and nanoparticles are observed in HAADF‐STEM images for PPhen‐bipy supported i) Ag^+^, j) Sn^4+^, k) Ir^3+^, and l) Au^3+^. Corresponded experimental and fitted EXAFS results are shown in a) [bipy‐FeCl_4_]^−^, b) [bipy‐CoCl_2_], c) [bipy‐Ni(H_2_O)_4_]^2+^, d) [bipy‐CuCl_2_], e) [bipy‐ZnCl_2_], f) [bipy‐RhCl_4_]^−^, g) [bipy‐PdCl_2_] and h) [bipy‐PtCl_4_], respectively. The coordination number and distance of M−Cl and M−N are listed in the Supporting Information, Table S3.

The homogeneity of the single sites is crucial to provide a key platform to investigate the structure–activity relationship.^[28]^ Here in the PPhen‐bipy system, the bidentate nitrogen coordinative environment is well preserved. Therefore, the formation of surface [bipy‐MX_*n*_] site is predictable according to coordination chemistry. We further extend the scope of such system to twelve metal cations based on their positions in the periodic table and their applications in catalysis. They are 3d metals, including Fe^3+^, Co^2+^, Ni^2+^, Cu^2+^, and Zn^2+^, noble metals, including Rh^3+^, Pd^2+^, Ag^+^, Ir^3+^, Pt^4+^, and Au^3+^, and p‐block element Sn^4+^. This selection covers cations with valence electrons from d^5^ to d^10^ and ionic radius from 69 pm to 129 pm, which can form tetrahedral, square planar and octahedral geometries that lead to different catalytic behaviours. [bipy‐FeCl_4_]^−^, [bipy‐CoCl_2_], [bipy‐CuCl_2_], [bipy‐ZnCl_2_], [bipy‐RhCl_4_]^−^, [bipy‐PdCl_2_], and [bipy‐PtCl_4_]^−^ are obtained with good metal dispersion over PPhen‐bipy, as determined in HAADF‐STEM and XAFS studies (Figure 4 a,b,d–h). XANES confirms that the oxidation states of metals are the same as the metal precursors (Supporting Information, Figure S23). Ni^2+^ forms [bipy‐Ni(H_2_O)_4_]^2+^ sites instead of [bipy‐NiCl_4_]^2−^ (Figure 4 c). 4d and 5d single sites are easily recognized in the HAADF‐STEM compared to 3d metals, due to the higher Z‐contrast of these heavy metal cations. The BET equivalent surface area of PPhen‐[bipy‐RhCl_4_]^−^ is 410 m^2^ g^−1^, similar to that of the PPhen‐[bipy‐Ru^III^Cl_4_]^−^ (Supporting Information, Table S4 and Figure S6). Ag, Sn, Ir, and Au form a majority of clusters instead of single sites (Figure 4 i–k), which is possibly due to the weak M−N/M−Cl coordination bond and the strong M−M or M−O bonds that promote cluster formation. Apart from metal cations, the selection of ligands is also controllable. [bipy‐RuCl_4_]^−^, [bipy‐RuBr_4_]^−^, [bipy‐CuCl_2_], and [bipy‐Cu(H_2_O)_4_]^2+^ are achieved accordingly (Supporting Information, Figure S24). The diverse selection of metal cations and ligands provides a single‐site palette with controllable geometric and electronic structures for a target catalytic reaction.

[bipy‐PtCl_4_]^−^ and [bipy‐CuCl_2_] sites are studied in the CO oxidation under the same condition to that of the Ru single sites. Both show very limited CO conversion (Supporting Information, Figure S25), despite that Pt and Cu single sites over metal oxide support are active in this reaction (Supporting Information, Table S9). Such a difference between molecularly designed Ru and Cu/Pt sites can shed light on the future research.

## Conclusion

We have designed an air‐stable *cis*(CO)‐*trans*(Cl)‐[bipy‐Ru^II^(CO)_2_Cl_2_] single site over a PPhen‐bipy framework with molecular‐level accuracy. Compared to the completely inert Ru^III^ site, the Ru^II^ site is active for CO oxidation owing to its unique electronic and geometric structure. Such a Ru^II^ single site can only be formed in the simultaneous Ru^III^ to Ru^II^ reduction by H_2_ and the ligand exchange from Cl^−^ to CO. With the help of a well‐defined pristine single site, such solid/gas interface coordination chemistry can be used to explain the surface bond breaking and formation at molecular level. The design method is generally applicable and is versatile for metal centers that are ubiquitous in catalysis, as supported by the synthesis of [bipy‐MX_*n*_] sites for another nine elements.

## Conflict of interest

The authors declare no conflict of interest.

## Supporting information

As a service to our authors and readers, this journal provides supporting information supplied by the authors. Such materials are peer reviewed and may be re‐organized for online delivery, but are not copy‐edited or typeset. Technical support issues arising from supporting information (other than missing files) should be addressed to the authors.

Supplementary
