# Supplementary material for: Design, Identification, and Evolution of a Surface Ruthenium(II/III) Single Site for CO Activation
Source: Angew Chem Int Ed Engl. 2020 Nov 13;60(3):1212–9. doi: 10.1002/anie.202008370 (PMC7839529; doi:10.1002/anie.202008370)
Supplement: Supplementary file 1 — Supplementary [file ANIE-60-1212-s001.pdf]

Angewandte Chemie
Eine Zeitschrift der Gesellschaft Deutscher Chemiker

Supporting Information

Design, Identification, and Evolution of a Surface Ruthenium(II/III) Single Site for CO Activation

*Liqun Kang, Bolun Wang, Adam Thetford, Ke Wu, Mohsen Danaie, Qian He, Emma K. Gibson, Ling-Dong Sun, Hiroyuki Asakura, C. Richard A. Catlow, and Feng Ryan Wang**

anie_202008370_sm_miscellaneous_information.pdf

SUPPORTING INFORMATION

Table of Contents

Experimental Procedures 2

Supporting Figures 5

Supporting Tables 19

References 25

Author Contributions 27

Experimental Procedures

Materials Synthesis

To prepare PPhen-bipy, 1,2,4,5-tetrabromobenzene (1.316 g, 3.34 mmol), 5,5'-dibromo-2,2'-bipyridine (0.342 g, 1.09 mmol), benzene-1,4-diboronic acid (1.290 g, 7.78 mmol), K_2CO_3 (2.0 M aqueous solution, 15 mL) and $Pd(PPh_3)_4$ (0.30 g, 0.26 mmol) were added into 120 mL dimethylformamide (DMF). The mixture was degassed through three freeze-pump-thaw cycles. The mixture was then purged with Ar and refluxed at 150 °C for 20 h under stirring. After the heating, the mixture was poured into 600 mL water under fierce stirring to make K_2CO_3 and DMF dissolve in water. The product was collected by filtration and was washed with water and methanol. Approximately 1 g of dark-green product was obtained in each batch. To remove the Pd in the crude product, the collected product was dispersed in 200 mL 30% H_2O_2 aq./37% HCl aq./H_2O/EtOH solution (volume ratio 1:5:5:10) and kept stirring at room temperature overnight and then heated at 60 °C for 4h. The bulk Pd cluster was oxidized to Pd^{2+} and dissolved into solution. After the heating, the product was collected by filtration and was washed with water and methanol, dried at 60 °C overnight. To further remove the Pd^{2+} coordinated with the bipyridine ligand, the 1 g of light-yellow product was dispersed in 200 mL 32% NH_3 aq./H_2O/EtOH solution (volume ratio 1:15:30) with 10 g $Na_2EDTA\cdot 2H_2O$ and kept stirring at 70 °C for 24h. After the heating, the product was collected by filtration and was washed with water and methanol. The final product was earthy yellow and dried at 60 °C in a vacuum oven overnight.

PPhen-[bipy-$RuCl_4$]H was synthesized *via* the following steps. 1.0 g of PPhen-bipy product obtained via the previous method and 150 mg $RuCl_3\cdot xH_2O$ were added into 250 mL round bottom flask with 50 mL 10% HCl aq. and 50 mL ethanol. The mixture was stirred at 60 °C for 5 hours, during this time the colour of solution changed from dark brown to reddish brown. Then the product was collected by filtration and washed with water and methanol until the filtrate become colourless. The final product was dried at 60 °C in a vacuum oven overnight.

To obtain the PPhen-bipy supported other metal catalysts, combination of different metal precursors and solutions should be used. $FeCl_3\cdot 6H_2O$, $CoCl_3\cdot 6H_2O$, $NiCl_2$, $CuCl_2\cdot 2H_2O$, $ZnCl_2$, $RhCl_3\cdot xH_2O$, $PdCl_2$, $SnCl_4\cdot 5H_2O$, $IrCl_3\cdot xH_2O$, H_2PtCl_6, and $HAuCl_4$ were used accompanied with 10% HCl aq./EtOH solution (volume ratio 1:1) for synthesis of PPhen-[bipy-$FeCl_4$]H, PPhen-[bipy-$CoCl_2$], PPhen-[bipy-$Ni(H_2O)_4$]Cl_2, PPhen-[bipy-$CuCl_2$], PPhen-[bipy-$ZnCl_2$], PPhen-[bipy-$RhCl_4$]H, PPhen-[bipy-$PdCl_2$], PPhen-[bipy-$PtCl_4$], PPhen-bipy-Ir, PPhen-bipy-Au and PPhen-bipy-Sn, respectively. Instead of Cl coordination, $CuSO_4\cdot 5H_2O$ in 10% H_2SO_4 aq., $RuBr_3\cdot xH_2O$ in 10% HBr aq. and $AgNO_3$ in 10% HNO_3 aq. were used for synthesis of PPhen-[bipy-$Cu(H_2O)_4$]SO_4, PPhen-[bipy-$RuBr_4$]H and PPhen-bipy-Ag respectively.

Calculation of theoretical bipy density in PPhen-[bipy-$RuCl_4$]H

1,2,4,5-tetrabromobenzene (Mw. = 393.70 $g\cdot mol^{-1}$, 1.316 g, 3.34 mmol), 5,5'-dibromo-2,2'-bipyridine (Mw. = 313.98 $g\cdot mol^{-1}$, 0.342 g, 1.09 mmol) and benzene-1,4-diboronic acid (Mw. = 165.75 $g\cdot mol^{-1}$, 1.290 g, 7.78 mmol) are used for synthesis of PPhen-bipy support. Such ratio of these precursors ensures the -Br/-$B(OH)_2$ ratio is equal to 1. We assume the Suzuki C-C coupling is 100% efficient, which stoichiometrically subtract all the -Br and -$B(OH)_2$ groups, leaving only the phenyl groups in the final product. Hence the yield of PPhen-bipy product is:

$$m_{\text{(PPhen-bipy)}} = \frac{1.316\text{ g}}{393.70\text{ g/mol}} \times 74.08\text{ g/mol} + \frac{0.342\text{ g}}{313.98\text{ g/mol}} \times 154.17\text{ g/mol} + \frac{1.290\text{ g}}{165.75\text{ g/mol}} \times 76.10\text{ g/mol} = 1.008\text{ g}$$

We assume that each Ru^{3+} is only coordinated with one bipy ligand. With 1.09 mmol of bipy ligands, the yield of PPhen-[bipy-$RuCl_4$]H is calculated as:

$$m_{\text{(PPhen-[bipy-RuCl}_4\text{]H)}} = 1.008\text{ g} + 1.09\text{ mmol} \times 243.89\text{ g/mol} = 1.274\text{ g}$$

The theoretical maximum loading of Ru (x_{max}) in the PPhen-[bipy-$RuCl_4$]H is calculated as:

$$x_{\text{max}} = \frac{1.09\text{ mmol} \times 101.07\text{ g/mol}}{1.274\text{ g}} \times 100\% = 8.6\text{ wt\%}$$

The theoretical maximum bipy loading amount in the PPhen-[bipy-$RuCl_4$]H (n_{max}) is calculated as:

$$n_{\text{max}} = \frac{1.09\text{ mmol}}{1.274\text{ g}} = 0.856\text{ mmol/g}$$

The ratio of actual Ru loading to the maximum bipy density is:

$$\frac{0.574\text{ mmol/g}}{0.856\text{ mmol/g}} \times 100\% = 67\%$$

The possible explanations for this 67% ratio are:

(1) The micro-structure of PPhen-bipy is made by random connections between phenyl groups and bipy groups, accordingly the distribution of bipy within the PPhen-bipy is homogeneous at macro level but inhomogeneous at micro level. Thus, the pore size and shape for each bipy ligand varies, and it is possible that some of the bipy ligands are inaccessible for the metal cations.

(2) The polyphenylene framework is hydrophobic, which to some extent will prevent the infiltration of the aqueous solution and inhibit the sufficient transportation of metal cations from liquid phase to solid phase.

(3) Not all the bipy ligands are converted into PPhen-bipy, leading to the decreased amount of actual bipy loading.

STEM and EDS study

High resolution aberration-corrected Annular Bright Field (ABF) and High Angle Annular Dark Field Scanning Transmission Electron Microscopy (HAADF-STEM) studies were performed at the electron Physical Science Imaging Centre (ePSIC). ABF and HAADF-STEM images were acquired simultaneously on probe-corrected (JEOL-COSMO) JEM ARM 300CF (JEOL, Japan) operated at 300 kV. All samples were prepared by sprinkling a small amount of dry powder on 400 mesh gold/copper grids with lacey carbon support film. A 30 μm probe-forming aperture was used, resulting in 22.4 mrad probe convergence semi-angle. A probe size of 8C was chosen to maximize the spatial resolution. The HAADF signal was gathered at 9.0 cm STEM camera length, integrating the scattered electron intensity between 77 and 209 mrad. In order to mitigate accumulation of carbon contamination during STEM imaging, the regions of interest were exposed to an intense electron “beam shower” for 15 min.

Energy-Dispersive X-ray Spectroscopy (EDS) results were obtained on the probe-corrected (CEOS) JEM ARM 200CF (JEOL, Japan) operated at 200 keV, which is equipped with large solid-angle dual EDX detectors for X-ray spectroscopy and elemental mapping. The EDX data acquisition was carried out in STEM imaging mode. Each EDX spectrum image is 80 × 80 pixels in size, with 0.1 second exposure time per pixel. The mapping procedure was performed with spatial drifting correction every 30 seconds. Gatan Microscopy Suite Software was used for EDS spectrum imaging data acquisition.

Ex situ and *in situ* XAFS measurements

X-ray Absorption Near Edge Structure (XANES) and extended X-ray Absorption Fine Structure (EXAFS) studies were performed at the Diamond Light Source (UK) on beamline B18[1]. Measurements were performed in transmission mode using ion chamber detectors through a QEXAFS setup with fast-scanning Si (111) (for K-edge of Fe, Co, Ni, Cu, Zn and L_3-edge of Pt) and Si (311) (for K-edge of Ru, Rh and Pd) double crystal monochromators. A couple of Pt-coated harmonic rejection mirrors were inserted between the monochromator and ion chamber to cut off the photons with higher energy (for K-edge of Fe, Co, Ni, Cu and Zn). Different metal foils were used for energy shift calibrations of each absorption edge. Metal oxides and chlorides were diluted with boron nitride and pressed into 8 mm diameter pellets as standard materials. The spectrum of each sample was measured 2 times and merged to improve the signal-noise ratio. The XAFS data of some standard materials ($RuCl_3$, $[Ru(NH_3)_6]Cl_2$, $[Ru(NH_3)_6]Cl_3$ and $[Ru(NH_3)_5Cl]Cl_2$) were utilized by SPring-8 BL14B2 XAFS standard sample database.

The *in situ* XAFS study of PPhen-[bipy-$RuCl_4$]H catalysts was performed in a plug-flow microreactor with same X-ray beam setting-up and data acquisition parameters. The catalysts powder was packed into a kapton foil tube (diameter 5 mm) with quartz wool at both ends. A controlled flow of mixed gases via 4 mass flow controllers (pure He, 1% CO in He, 2% H_2 in He and 5% O_2 in He, respectively) was introduced into the reaction tube at ambient pressure. A hot air gun was placed under the reaction tube to heat the catalysts bed (heating and cooling ramp rate of 10 °C·min^{-1} and 20 °C·min^{-1}, respectively). The heating zone was sheathed with a ceramic drivepipe to improve the heat conductivity and prevent non-uniform heating. There are two 3 mm × 15 mm windows on both side of the ceramic drivepipe to let X-rays passing through. A K-type thermal couple was positioned inside the catalysts bed to monitoring the temperature. During the reaction, XAFS data was acquired continuously with spectra obtained every 135 seconds (k_{max} = 17, step size 0.3 eV). The outlet gas composition was analysed using a Quadrupole Mass Spectrometer Quantitative Gas Analyser (Hiden Analytical, UK). The Hiden QGA could continuously sampling and scan atomic mass range from 1 to 200 AMU with 500 times/second for measurement speed. The analysis sensitivity is 100% to 100 PPB subject to spectral interference. The gas or vapour spectrum were simulated and analysed by automatic subtraction of spectral overlaps.

XAFS study of Cl K-edge and Ru $L_{3,2}$-edge were conducted at BL6N1 beamline of Aichi Synchrotron Radiation Centre (Aichi Science & Technology Foundation, Japan). Soft X-ray beam was introduced via InSb (111) monochromator to a surface XAFS experiment chamber equipped with an electrostatic hemispherical photoelectron spectrometer (SPECS PHOIBOS 150). XAFS data were acquired in both conversion electron yield (CEY) mode and partial fluorescence yield (PFY) mode with energy range of 2730-3150 eV at 0.2 eV step size. Ru foil and RuO_2 were measured as standard reference for energy calibration.

XAFS data was analysed by Demeter (including Athena and Artemis methods, version 0.9.25)[2]. Athena software was used for data extraction and XANES analysis. Artemis software was used to fit the Ru K-edge EXAFS data (fitting range 3.3 Å^{-1} < k < 13.7 Å^{-1} and 1.0 Å < R < 3.5 Å). The amplitude reduction factor S_0^2 for each edge was calculated from EXAFS analysis of the metal foil and used as fixed parameter for EXAFS analysis.

DRIFTS, ATR-FTIR and Far-FTIR study

Diffuse reflectance infrared Fourier transform spectroscopy (DRIFTS) data were collected on an Agilent Carey 680 FTIR spectrometer (Agilent, UK) equipped with liquid nitrogen cooled MCT detector and Harrick reaction chamber. PPhen-[bipy-$RuCl_4$]H powder was filled in a sample cup and placed inside the reaction chamber. The IR beam was directed into it by the Praying Mantis accessory. The time resolution of each spectrum is 60s (400–4,000 cm^{-1}), while 64 scans were taken and merged with a resolution of 4 cm^{-1} per minutes. The outlet gas was analysed by the same Hiden QGA mass spectrometer.

Attenuated Total Reflection Infrared (ATR-IR) spectroscopy was measured with Bruker ALPHA FTIR spectrometer. The spectra were taken within 4000-400 cm^{-1} range.

Far Fourier transform infrared spectroscopy (Far-FTIR) was measured with ThermoFisher Nicolet iS50 FTIR spectrometer. The spectra were taken within 1800-180 cm^{-1} range.

Other characterisations

Powder X-ray diffraction (PXRD) measurement was performed using Bruker D8 diffractometer with a voltage of 40 kV, at 30 mA, using a Cu source with $K_{\alpha1}$ = 1.540562 Å and $K_{\alpha2}$ = 1.544398 Å. The contributions of $K_{\alpha2}$ line in the XRD patterns were subtracted.

Nitrogen adsorption–desorption isotherms were recorded at 77 K using a Micromeritics 3Flex surface characterization analyser. The samples were degassed in vacuum at 200 °C overnight for removal of any contaminates. Specific surface areas were determined according to the BET model.

X-ray photoelectron spectrum (XPS) analysis were performed on a Thermo-scientific XPS K-alpha surface analysis machine using an Al source. The sample powder was immobilized on silicon chips for measurement. C 1*s* electron at 284.8 eV was used as standard reference to calibrate the photoelectron energy shift. XPS spectra in C 1*s* and Ru 3*d* region from 270 eV to 300 eV was collected (0.1 eV step size) and deconvoluted to identify the oxidation states of Ru. All the data analysis was performed on the CasaXPS software (version: 2.3.18PR1.0).

Thermogravimetry and Differential Scanning Calorimetry (TG-DSC) were used to measure the temperature-dependent mass changes and energetic effects. The experiment was carried out on STA 449 F5 Jupiter simultaneous thermal analyser (NETZCH, Germany). The thermal stability tests of PPhen-[bipy-$RuCl_4$]H and PPhen-bipy in synthetic air and argon were performed. About 5 mg of sample powder was placed into a Pt-Rh crucible (with pierced lids) for the measurement. A gas flow of 70mL/min was employed for either synthetic air or argon (protective gas: 20mL/min & purge gas: 50mL/min). The temperature range was set from R.T. to 1000 °C with a ramp speed of 10 °C/min. The temperature was monitored via a type S thermocouple.

Computational Methods

The simulated structures were based on a simplified octahedral model complex: a bipyridine (bipy) with the two nitrogen atoms co-ordinated to the Ru and Ru further co-ordinated to CO and Cl. Calculations were done in Gaussian09[3] using the B3LYP[4] functional and LANL2DZ basis set for Ru and 6-31G(d,p) basis set for all other elements.

Supporting Figures

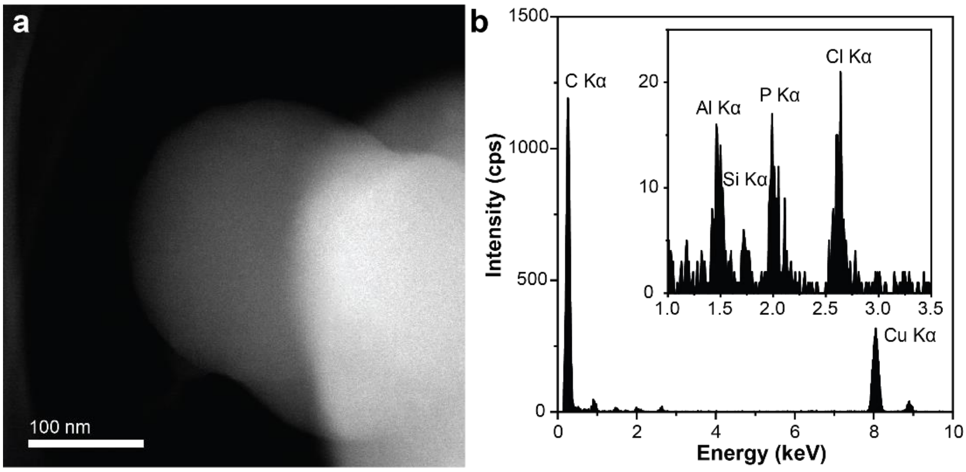

Figure S1. HAADF-STEM and EDS of PPhen-bipy. (a) No Pd clusters or nanoparticles were found in HAADF-STEM image of PPhen-bipy polymer (b) EDS of the same region shows that no X-ray emission from Palladium (Lα at 2.84 keV) was found for PPhen-bipy.

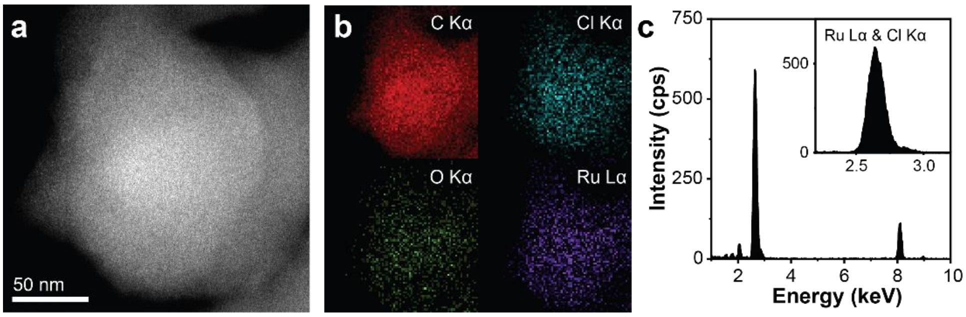

Figure S2. HAADF-STEM and EDS element mapping of PPhen-[bipy-$RuCl_4$]H. (a) HAADF-STEM image of PPhen-[bipy-$RuCl_4$]H. (b) EDS element mapping of C Kα, O Kα, Cl Kα and Ru Lα at the same region. (c) EDS summary of the specified area. There was an overlap Cl (Kα at 2.60 keV) and Ru (Lα at 2.56 keV) emissions. No obvious Pd Lα signal could be observed. For EDS, Limits of detection depend on the element, detector profiles and data acquisition time. The typical detection limit of EDS is less than 1000 ppm (0.1wt%) and could reach as low as 200 ppm (0.02 wt%) in the absence of peak interference.[5]

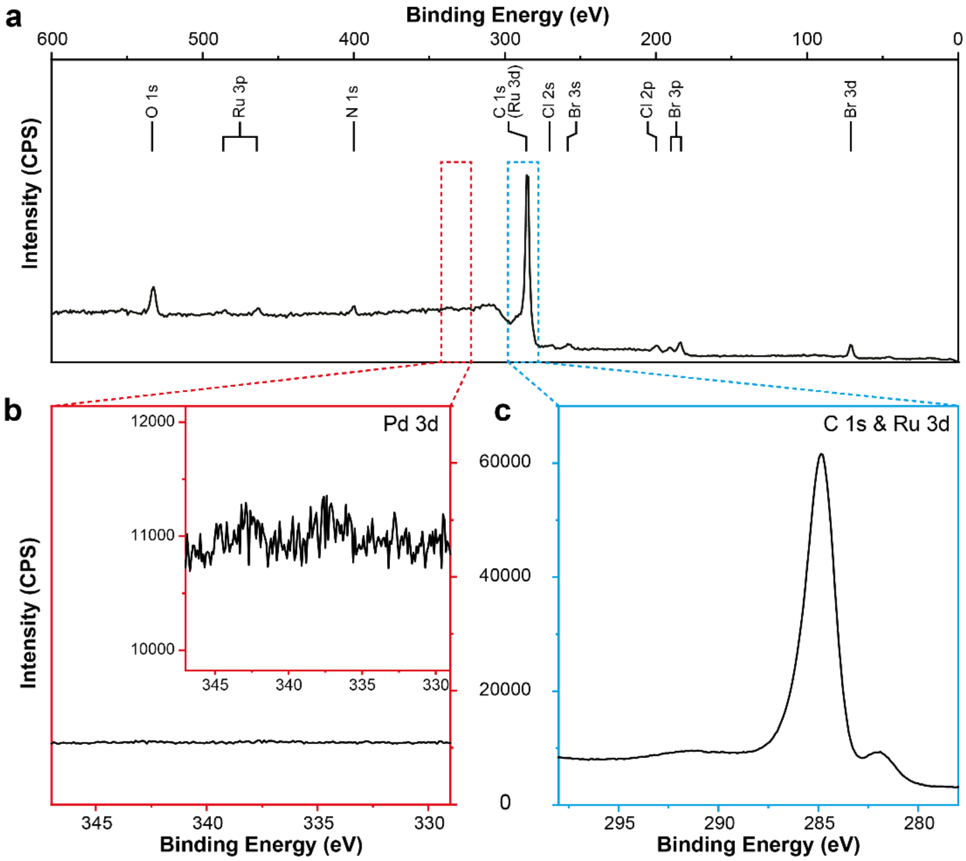

Figure S3. XPS spectra of PPhen-[bipy-$RuCl_4$]H. (a) Survey scan shows the presence of C, Ru, Br, Cl, N and O in the sample. (b) XPS fine scan at Pd 3*d* region. There is no obvious Pd 3*d* signals in the XPS spectra, suggesting the Pd concentration is lower than the typical detection limits of XPS (0.1 atom%).[6] (c) XPS fine scan at C 1*s* and Ru 3*d* region. The C 1*s* peaks are overlapping with Ru 3*d* peaks due to the similar binding energies.

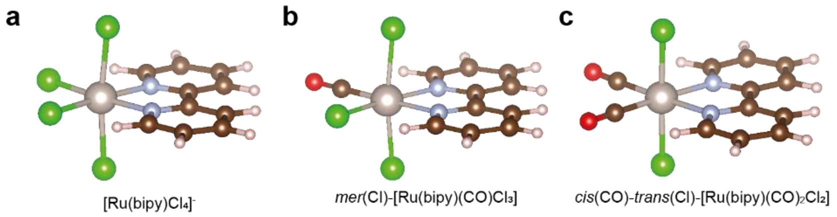

Figure S4. DFT simulated structure of $[Ru(bipy)Cl_4]^-$, *mer*(Cl)-$[Ru(bipy)(CO)Cl_3]$ and *cis*(CO)-*trans*(Cl)-$[Ru(bipy)(CO)_2Cl_2]$. DFT simulated structures of (a) $[Ru(bipy)Cl_4]^-$. (b) *mer*(Cl)-$[Ru(bipy)(CO)Cl_3]$ and (c) *cis*(CO)-*trans*(Cl)-$[Ru(bipy)(CO)_2Cl_2]$ (Ru is grey, Cl is green, O is red, C is brown, N is blue and H is white).

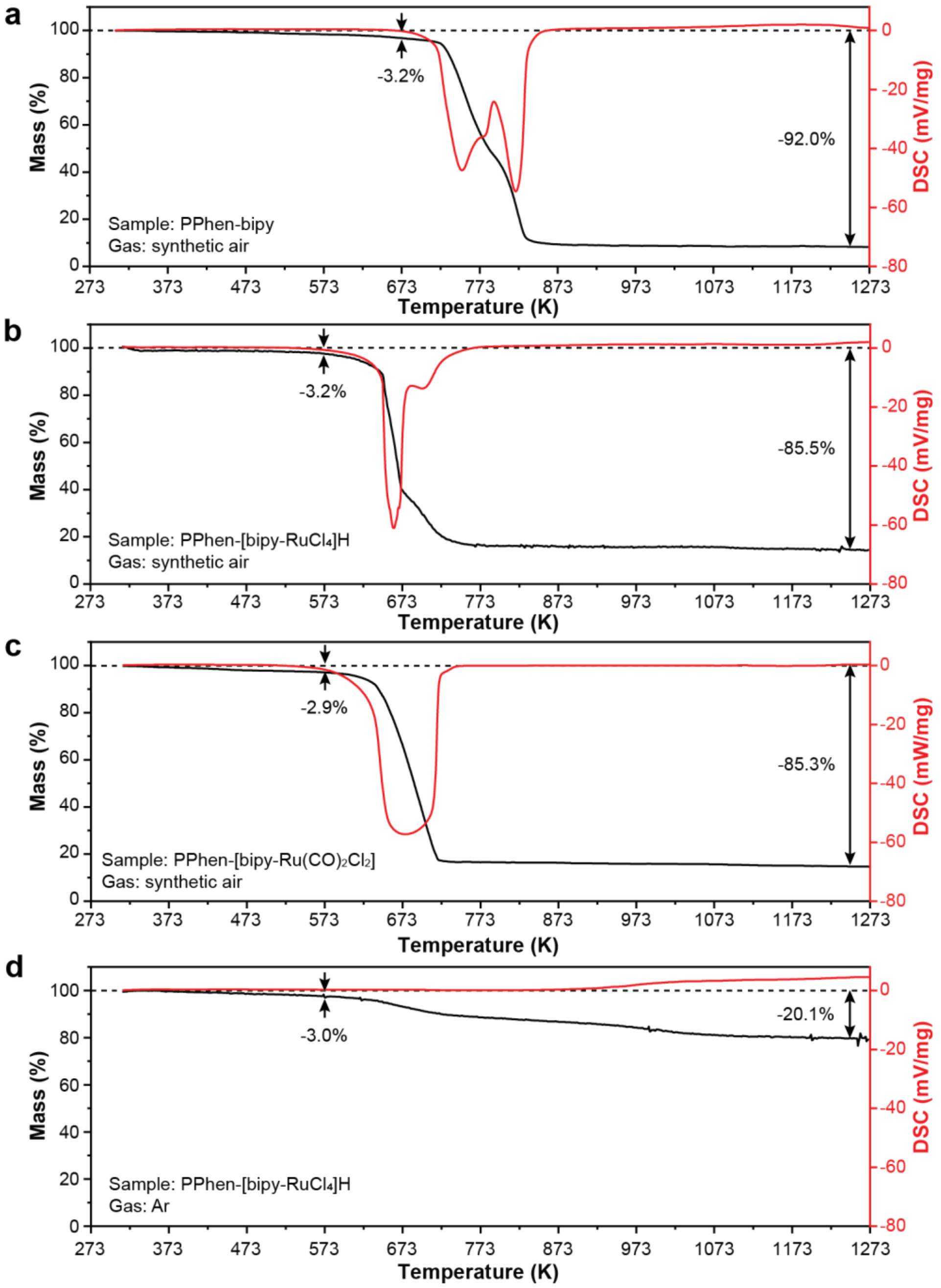

Figure S5. Temperature-dependent mass change and heat flow curve of PPhen-bipy, PPhen-[bipy-$RuCl_4$]H and PPhen-[bipy-$Ru(CO)_2Cl_2$]. (a) For PPhen-bipy, the TG curve showed slight mass loss of 3.2% at 400 °C, which was caused by the release of residual moisture. At peak temperatures of 474°C and 546 °C, distinctive overlapped exothermic effects were detected. The origin of these pronounced effects is due to the combustion of the organic content within the sample. At above 600 °C, neither the DSC signal nor the TG curve showed significant changes with only residual of 8.0% from polymer framework. (b) PPhen-[bipy-$RuCl_4$]H was stable in synthetic air until 300 °C with only 3.2% weight loss. The DSC peak was found at 389 °C, suggesting a decreased combustion temperature compares with PPhen-bipy. PPhen-[bipy-$RuCl_4$]H was fully decomposed above 700 °C with 14.5% residual. (c) For PPhen-[bipy-$Ru(CO)_2Cl_2$] in synthetic air, the TG curve showed slight mass loss of 2.9% at 300 °C, which was caused by the release of residual moisture. Exothermic effects were detected at peak temperature of 404 °C, resulting from the combustion of the organic content within the sample. At above 500 °C, neither the DSC signal nor the TG curve showed significant changes with only residual of 14.7% from polymer framework. (d) PPhen-[bipy-$RuCl_4$]H was stable in Ar , and the TG curve showed slight mass loss of 3.0% at 573 K. The similar weight loss percentages at 573 K in both synthetic air and argon were caused by the release of residual moisture, suggesting that the O_2 is not able to replace Cl^- ligands in PPhen-[bipy-$RuCl_4$]H even at high temperature. In summary, the calculated Ru loading amount is estimated to be 5.8wt%, based on the hypothesis that the extra residual was RuO_2. The calculation process of Ru loading (x) is as follows:

$$m_{(residual)} = m_{(PPhen\text{-}[bipy\text{-}RuCl4]H)} \cdot 14.5\% = m_{(PPhen\text{-}[bipy\text{-}RuCl4]H)} \cdot x_{(Ru\ loading)} \cdot Mw_{(RuO2)}/Aw_{(Ru)} + m_{(PPhen\text{-}bipy)} \cdot 8.0\%$$

$$14.5\% = x_{(Ru\ loading)} \cdot Mw_{(RuO2)}/Aw_{(Ru)} + \cdot (1-x_{(Ru\ loading)} \cdot (Mw_{(RuCl3)} + Mw_{(HCl)})/Aw_{(Ru)}) \cdot 8.0\%$$

$$x = (14.5\%-8.0\%)/(133.07/101.07-8.0\% \cdot (207.42+36.46)/101.07) = 5.8\%$$

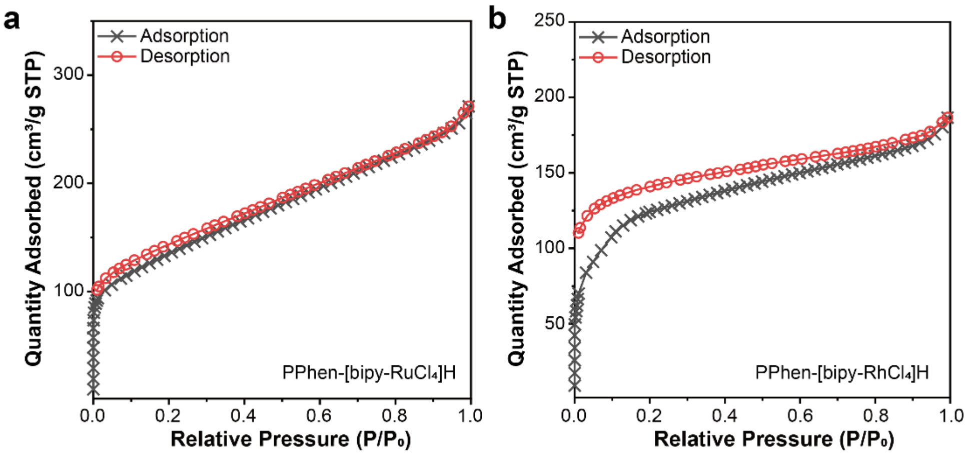

Figure S6. N_2 physisorption of PPhen-[bipy-RuCl$_4$]H and PPhen-[bipy-RhCl$_4$]H. The BET analysis results are shown in Table S4.

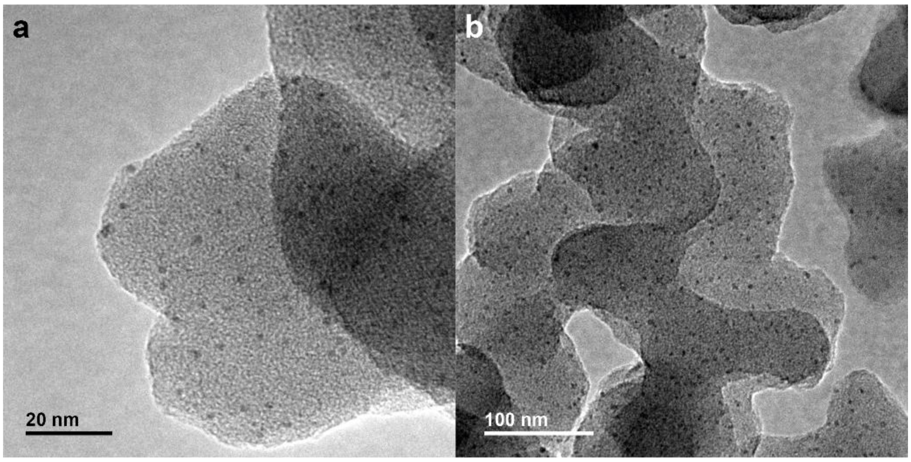

Figure S7. TEM image of PPhen-[bipy-Ru(III)Cl$_4$] after reduction in 15 vol% H_2 at 523K. Formation of Ru nanoparticles is clearly observed.

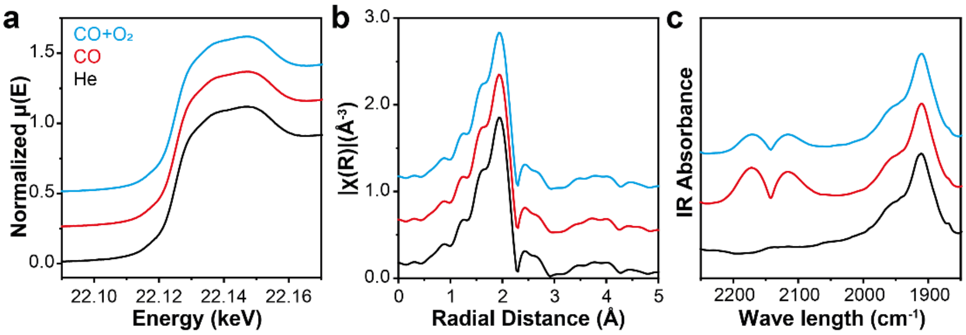

Figure S8. XANES, EXAFS and DRIFTS spectra of -[bipy-RuCl$_4$]$^-$ under pure He, CO and CO+O_2 at room temperature. (a) XANES spectra and (b) EXAFS spectra remain the same under pure He, CO and CO+O_2 atmosphere. (c) DRIFTS show only gas phase CO was detected.

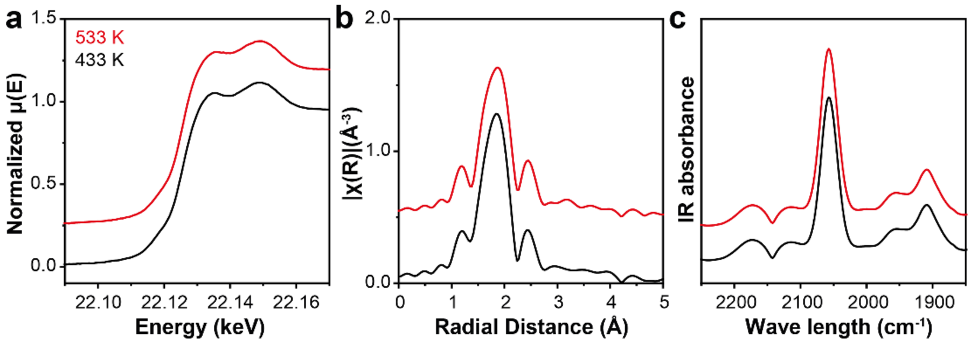

Figure S9. XANES, EXAFS and DRIFTS spectra of *mer*(Cl)-[bipy-Ru(CO)Cl_4] at 433K and 533K in 1%CO + 5%O_2. (a) XANES spectra, (b) EXAFS spectra and (c) DRIFTS spectra show there are no significant changes between 433K and 533K under 1%CO + 5%O_2 atmosphere.

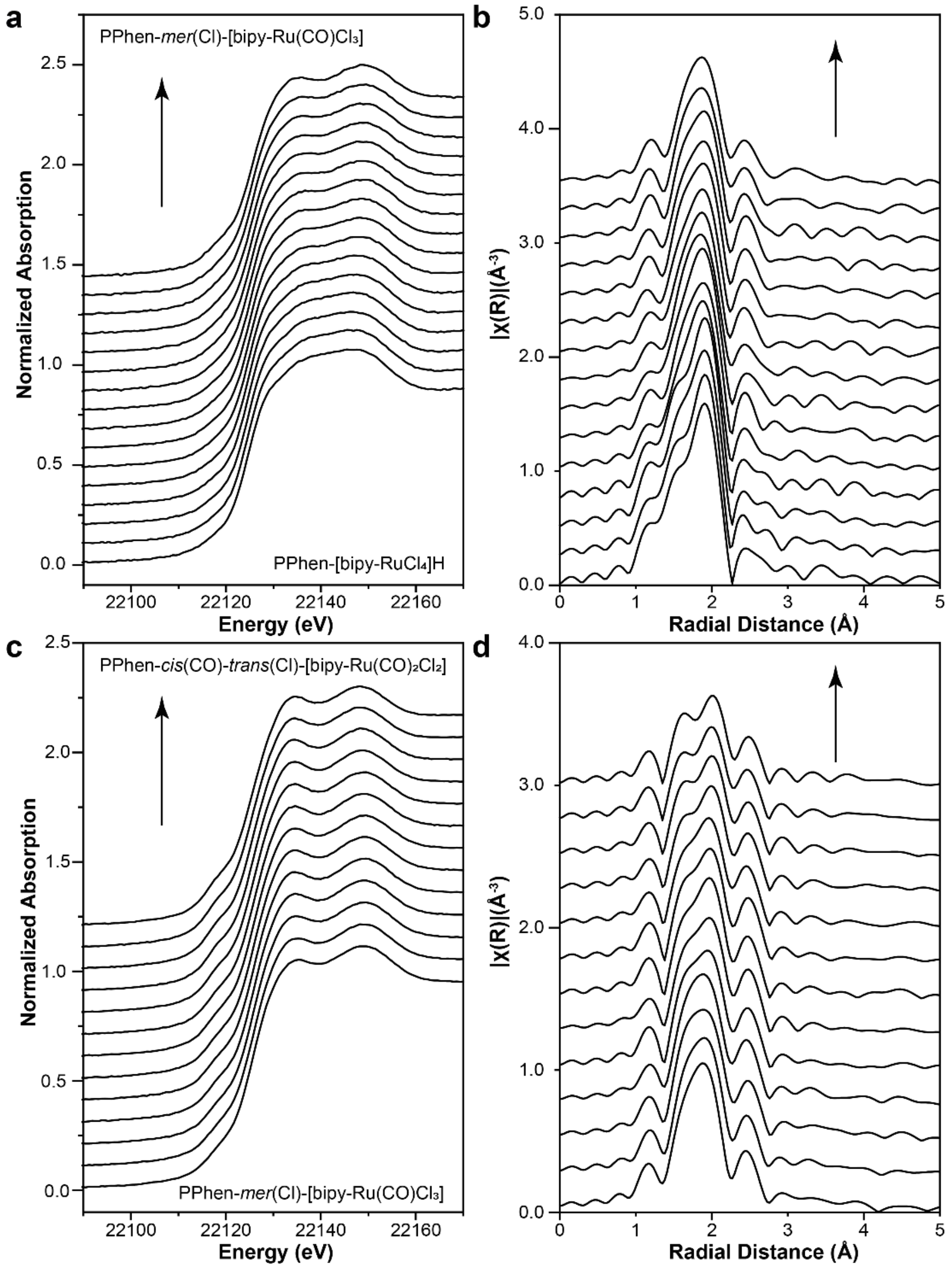

Figure S10. *In situ* XANES and k^2-weighted EXAFS of evolution from -[bipy-$RuCl_4$]$^-$ to *mer*(Cl)-[bipy-Ru(CO)Cl_3] and evolution from *mer*(Cl)-[bipy-Ru(CO)Cl_3] to *cis*(CO)-*trans*(Cl)-[bipy-Ru$(CO)_2Cl_2$]. (a) *In situ* XANES spectra of evolution from -[bipy-$RuCl_4$]$^-$ to *mer*(Cl)-[bipy-Ru(CO)Cl_3]. (b) *In situ* EXAFS spectra of evolution from -[bipy-$RuCl_4$]$^-$ to *mer*(Cl)-[bipy-Ru(CO)Cl_3]. (c) *In situ* XANES spectra of evolution from *mer*(Cl)-[bipy-Ru(CO)Cl_3] to *cis*(CO)-*trans*(Cl)-[bipy-Ru$(CO)_2Cl_2$]. (d) *In situ* EXAFS spectra of evolution from *mer*(Cl)-[bipy-Ru(CO)Cl_3] to *cis*(CO)-*trans*(Cl)-[bipy-Ru$(CO)_2Cl_2$].

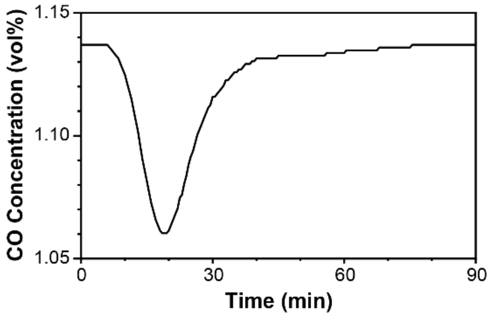

Figure S11. CO titration of PPhen-[bipy-$RuCl_4$]H. 1% CO in N_2 with a flow rate of 100 mL·min^{-1} was introduced to 100 mg PPhen-[bipy-$RuCl_4$]H powder at 433K. The weight hourly space velocity (WHSV) is 60,000 ml·h^{-1}·g^{-1}. 1.25 mL CO is consumed during the CO titration (integrated area). The estimated Ru loading concentration is 5.6wt% based on this value, which is in good agreement with 5.8wt% Ru loading calculated from the TGA in fig. S4. Calculation process of Ru loading (*y*) based on CO titration is as follows:

$$n_{(CO)} = V_{(CO)} / V_m = m_{(PPhen\text{-}[bipy\text{-}RuCl4]H)} \cdot y / A_{w(Ru)} = n_{(Ru)}$$

$$1.25\text{mL} / (22.4\text{L}\cdot\text{mol}^{-1}) = 100\text{ mg} \cdot y / (101.07\text{g}\cdot\text{mol}^{-1})$$

$$y = 5.6\%$$

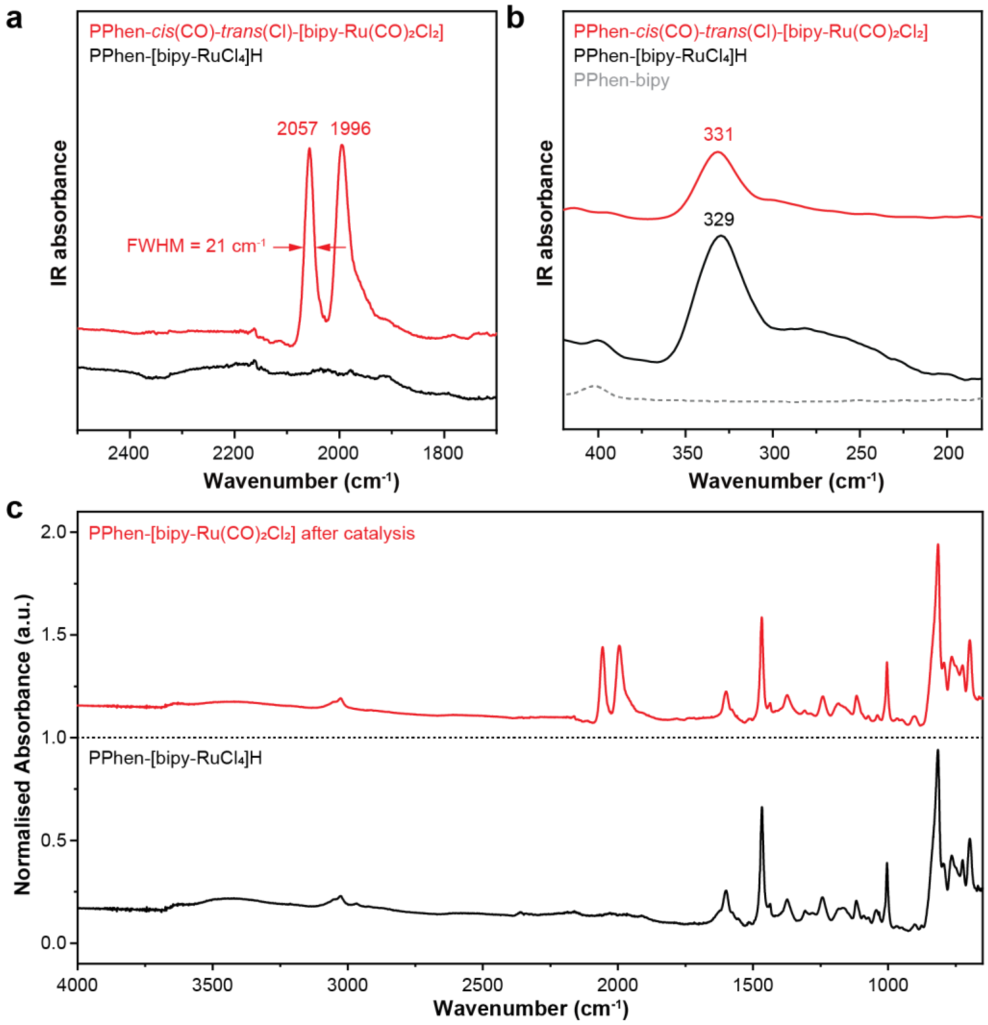

Figure S12. *Ex situ* ATR-FTIR and far-FTIR results. (a) ATR-FTIR spectra of PPhen-[bipy-$RuCl_4$]H (black curve) and PPhen-*cis*(CO)-*trans*(Cl)-[bipy-$Ru(CO)_2Cl_2$] (red curve). The absorption peaks at 2057 and 1996 cm^{-1} are corresponded to stretch vibration of terminal coordinated CO. The measured FWHM is 21 cm^{-1}, which is within the range CO in the molecular complex. (b) Far-FTIR spectrum of PPhen-bipy (grey curve), PPhen-[bipy-$RuCl_4$]H (black curve) and PPhen-*cis*(CO)-*trans*(Cl)-[bipy-$Ru(CO)_2Cl_2$] (red curve). These absorption peaks around 330 cm^{-1} are attributed to the Ru-Cl bond stretch vibration. (c) Full range FTIR spectra of PPhen-[bipy-$Ru(CO)_2Cl_2$] after catalysis and fresh PPhen-[bipy-$RuCl_4$]H. Except for the additional FTIR absorption between 2100 to 1900 cm^{-1} by terminal coordinated CO, there is no other significant changes in FTIR spectra, which suggest the PPhen-bipy framework remained unchanged after catalysis.

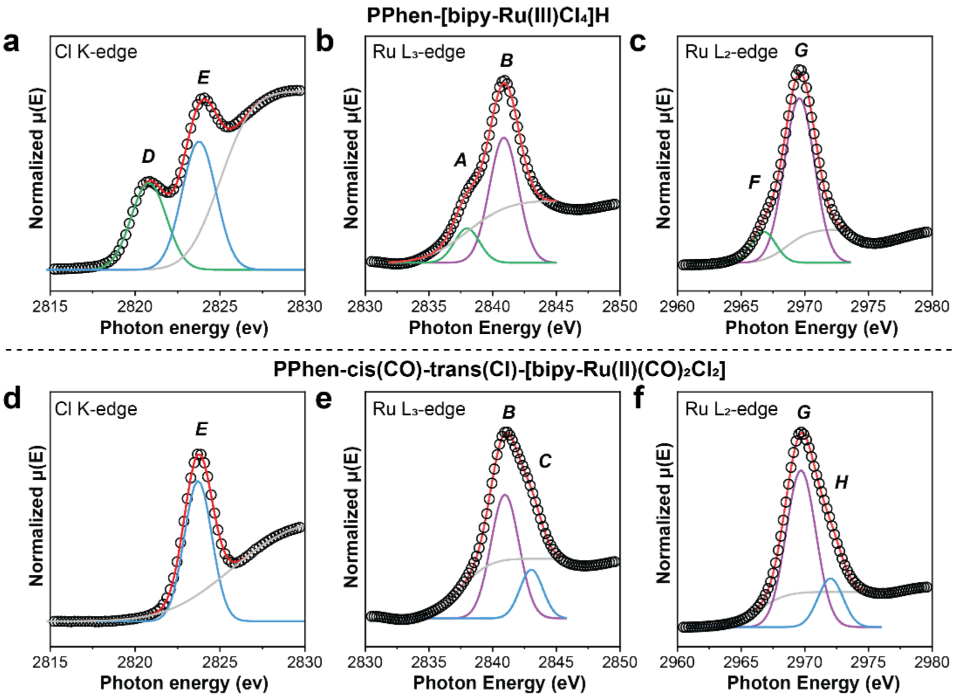

Figure S13. Peak fitting profile of Ru L_3-edge, Ru L_2-edge and Cl K-edge XANES of PPhen-[bipy-Ru(III)Cl_4]H and PPhen-*cis*(CO)-*trans*(Cl)-[bipy-Ru(II)$(CO)_2Cl_2$]. (a) Cl K-edge, (b) Ru L_3-edge and (c) Ru L_2-edge XANES of PPhen-[bipy-Ru(III)Cl_4]H. (d) Cl K-edge, (e) Ru L_3-edge and (f) Ru L_2-edge XANES of PPhen-*cis*(CO)-*trans*(Cl)-[bipy-Ru(II)$(CO)_2Cl_2$]. The peak fitting results are listed in Table S6.

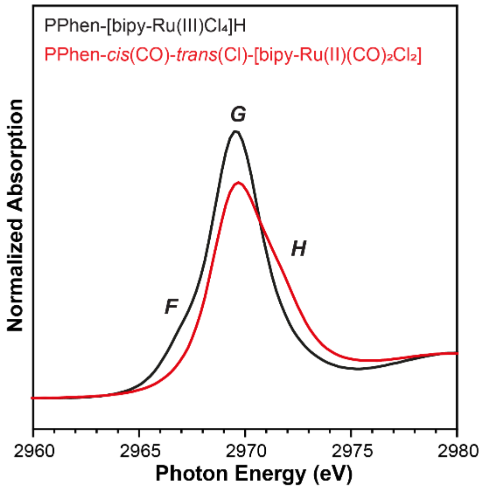

Figure S14. Ru L_2-edge XANES of PPhen-[bipy-Ru(III)Cl_4]H and PPhen-*cis*(CO)-*trans*(Cl)-[bipy-Ru(II)$(CO)_2Cl_2$]. The investigation at Ru L_2-edge shows consistent results as Ru L_3-edge in Figure 3. Peak *F*, *G* and *H* are attributed to Ru $2p_{1/2} \rightarrow t_{2g}$, Ru $2p_{1/2} \rightarrow e_g$ and Ru $2p_{1/2} \rightarrow$ CO π*transition respectively. The peak *F* of $2p_{1/2} \rightarrow t_{2g}$ transitions are dipole forbidden in octahedral complexes, so it has been less pronounced compares to peak *A* at Ru L_3-edge.[7] For Ru-bipy complex, it has been found that Ru $2p \rightarrow$ bipy π* transitions exhibit nearly the same energy levels as e_g orbitals, so it will overlap with peak *B* and *G* in both L_3 and L_2 edges.[7-8]

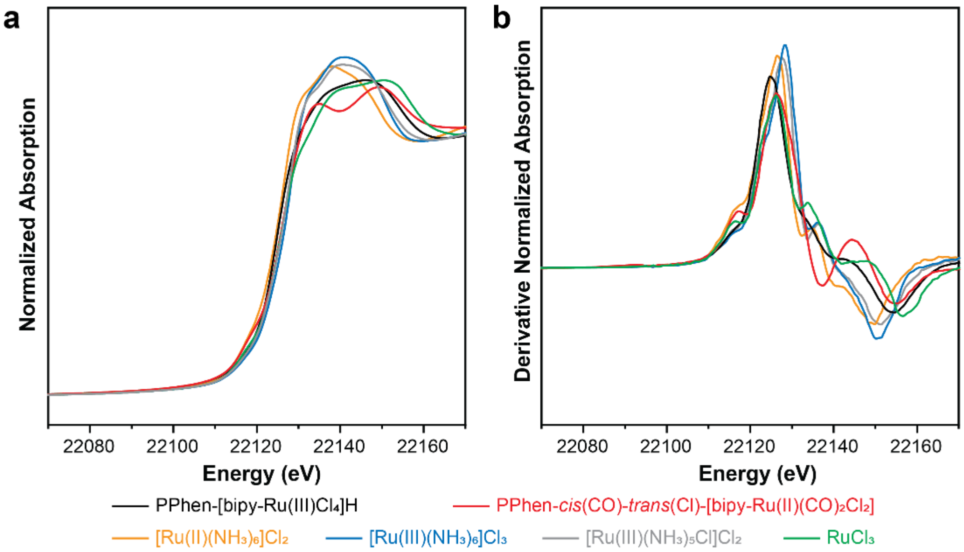

Figure S15. Normalized XANES and derivative XANES spectra at Ru K-edge of Ru reference materials. (a) XANES and (b) derivative XANES spectra of PPhen-[bipy-$RuCl_4$]H (black curve), PPhen-*cis*(CO)-*trans*(Cl)-[bipy-Ru(II)$(CO)_2Cl_2$] (red curve), $[Ru(NH_3)_6]Cl_2$ (orange curve), $[Ru(NH_3)_6]Cl_3$ (dark blue curve), $[Ru(NH_3)_5Cl]Cl_2$ (grey curve) and $RuCl_3$ (green curve). The edge position values are listed in Table S7. The shift of Ru K-edge position is strongly affected not only by oxidation states of Ru but also ligands and coordination structures.[9] The energy shift of Ru K-edge XANES is proportional to the oxidation state of Ru to a certain extent, but this is not the only dominant factor. The XANES features also contribute from the electron configurations, coordination geometries and ligand types. Such approach which to determine the oxidation states just based on the edge shift is priori limited to compounds with similar electronic configuration, local coordination and geometry.

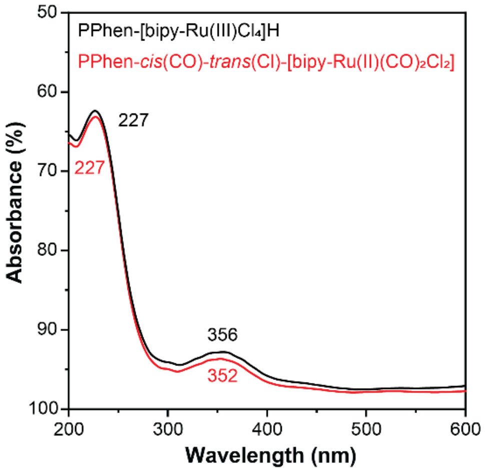

Figure S16. UV-Vis spectra of PPhen-[bipy-$RuCl_4$]H and PPhen-*cis*(CO)-*trans*(Cl)-[bipy-Ru(II)$(CO)_2Cl_2$]. The absorption peaks at 227nm and 352/356 nm are attributed to π→π* transition in aromatic groups and *d-d* transition in Ru single sites respectively.

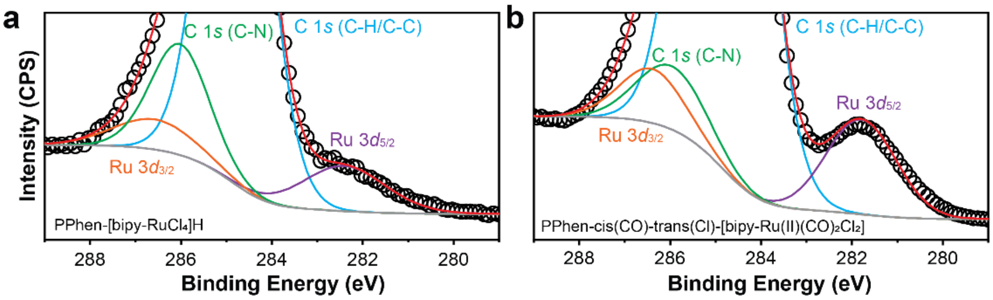

Figure S17. XPS Peak fitting results of PPhen-[bipy-$RuCl_4$]H and PPhen-[bipy-Ru$(CO)_2Cl_2$] at C 1*s* and Ru 3*d* region. Peaks of photoelectrons excited from Ru $3d_{5/2}$ (Violet curve), Ru $3d_{3/2}$ (orange curve) and C 1*s* (blue curve for C-C/C-H and green curve for C-N) were deconvoluted from the original data. The fitting results are listed in Table S8.

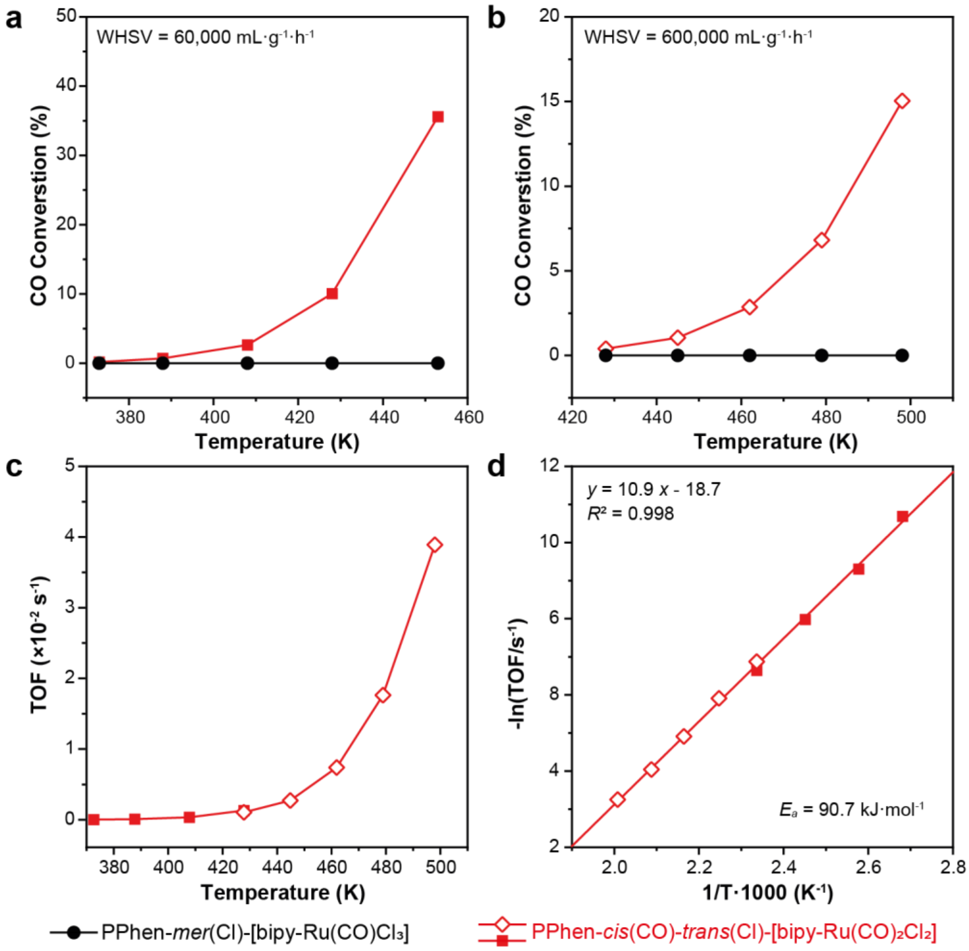

Figure S18. The conversion of CO during oxidation as a function of reaction temperature and plot of TOF as a function of reaction temperature. The conversion of CO during oxidation at (a) WHSV = 60,000 ml·h^{-1}g^{-1} (1% CO, 5% O_2, and N_2 balancing) and (b) WHSV = 600,000 ml·h^{-1}g^{-1} (2.0% CO, 2.5% O_2, and N_2 balancing). (c) Plot of TOF as a function of temperature. (d) Arrhenius plot showing E_a = 90.7 kJ·mol^{-1}. The TOF values are determined based on the following assumptions: (1) All the Ru are in the form of PPhen-[bipy-Ru(CO)$_2$Cl$_2$]; (2) All the PPhen-[bipy-Ru(CO)$_2$Cl$_2$] sites are identically active for the reaction and identically accessible for CO/O_2 gas molecules; (3) No CO/O_2 gas diffusion limitation within the micropore and mesopores. An example of how TOF value is calculated is shown below:

For WHSV = 60,000 ml·h^{-1}g^{-1} (1% CO, 5% O_2, and N_2 balancing), the conversion of CO at 428 K is 10.04 %. Therefore, the TOF value is:

$$\mathrm{TOF_{428K}} = \frac{\text{Molecules of CO converted per second}}{\text{Number of Ru sites}} = \frac{60{,}000\ \mathrm{mL{\cdot}h^{-1}{\cdot}g^{-1}} \times 1.0\ \% \times 10.04\ \% \ \times 101.07\ \mathrm{g{\cdot}mol^{-1}}}{22.4\ \mathrm{L{\cdot}mol^{-1}} \times 5.8\ \mathrm{wt\%}} = \frac{7.47 \times 10^{-7}\ \mathrm{s^{-1}}}{5.74 \times 10^{-4}} = 0.13 \times 10^{-2}\ \mathrm{s^{-1}}$$

For WHSV = 600,000 ml·h^{-1}g^{-1} (2.0% CO, 2.5% O_2, and N_2 balancing), the conversion of CO at 479 K is 6.82 %. Therefore, the TOF value is:

$$\mathrm{TOF_{479K}} = \frac{\text{Molecules of CO converted per second}}{\text{Number of Ru sites}} = \frac{600{,}000\ \mathrm{mL{\cdot}h^{-1}{\cdot}g^{-1}} \times 2.0\ \% \times 6.82\ \% \ \times 101.07\ \mathrm{g{\cdot}mol^{-1}}}{22.4\ \mathrm{L{\cdot}mol^{-1}} \times 5.8\ \mathrm{wt\%}} = \frac{1.01 \times 10^{-5}\ \mathrm{s^{-1}}}{5.74 \times 10^{-4}} = 1.77 \times 10^{-2}\ \mathrm{s^{-1}}$$

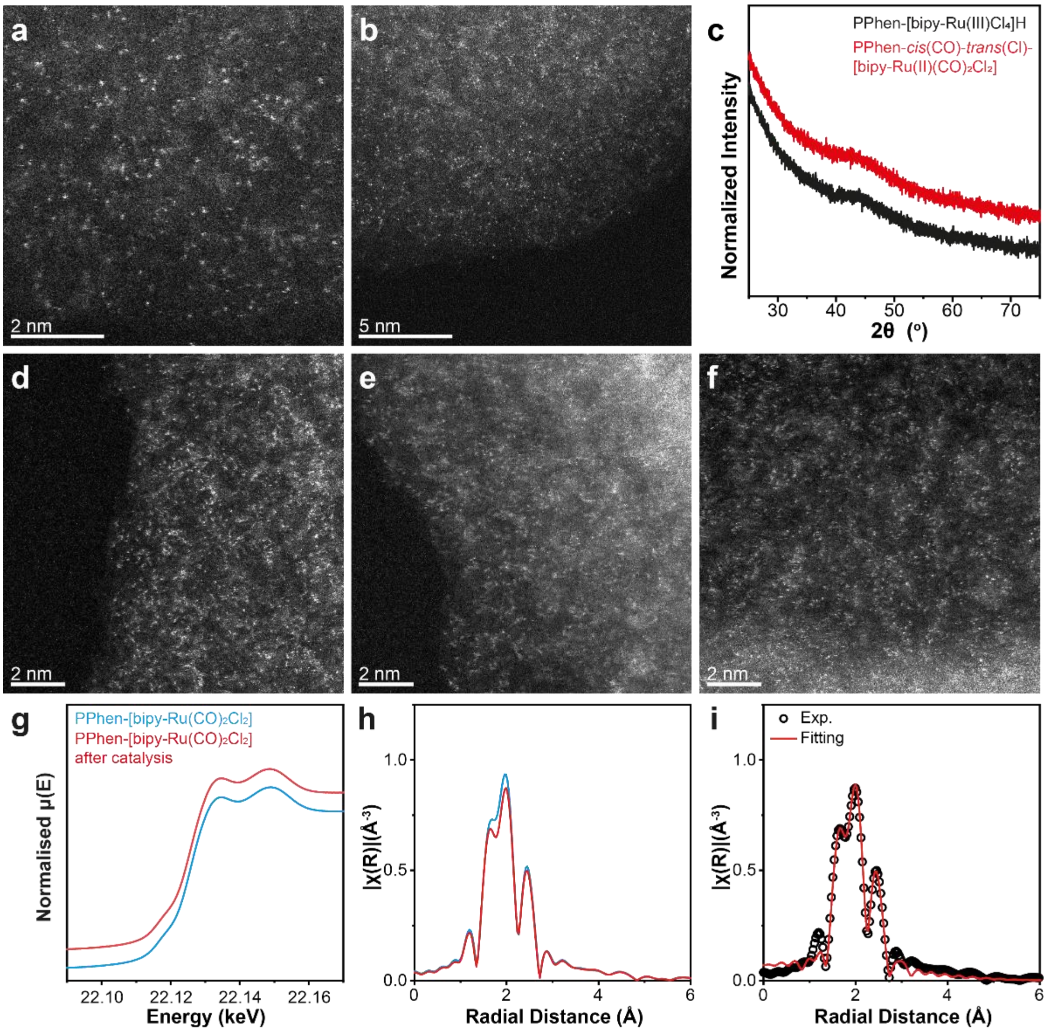

Figure S19. HAADF-STEM image, XRD pattern and XAFS of *cis*(CO)-*trans*(Cl)-[bipy-Ru(II)(CO)$_2$Cl$_2$] after catalysis. (a, b) HAADF-STEM image of *cis*(CO)-*trans*(Cl)-[bipy-Ru(II)(CO)$_2$Cl$_2$], showings the distribution of Ru single-sites with no formation of Ru clusters. (c) XRD patterns of PPhen-[bipy-RuCl$_4$]H and PPhen-*cis*(CO)-*trans*(Cl)-[bipy-Ru(II)(CO)$_2$Cl$_2$]. (d, e, f) HAADF-STEM image of *cis*(CO)-*trans*(Cl)-[bipy-Ru(II)(CO)$_2$Cl$_2$] after catalysis, showings the distribution of Ru single-sites with no formation of Ru clusters. Ru K-edge (g) XANES, (h) EXAFS and (i) EXAFS fitting results of PPhen-[bipy-Ru(II)(CO)$_2$Cl$_2$] after catalysis.

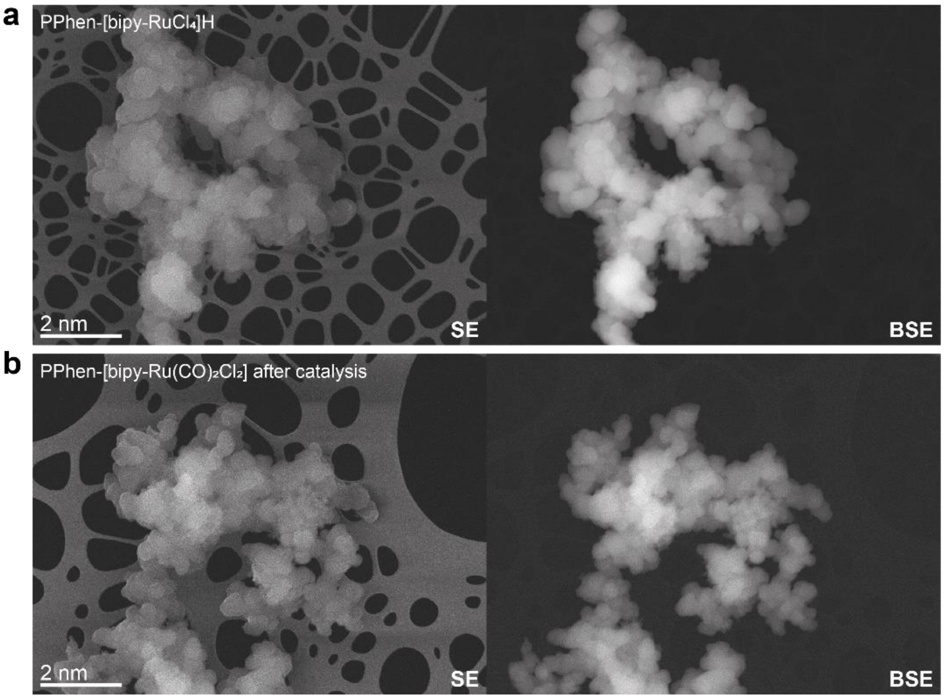

Figure S20 Scanning Electron Microscopy image of (a) fresh PPhen-[bipy-$RuCl_4$]H and (b) PPhen-[bipy-$Ru(CO)_2Cl_2$] after catalysis: Secondary Electrons Image (left) and Backscattered Electrons Image (right). The morphology of polymer particles of PPhen-[bipy-$Ru(CO)_2Cl_2$] after catalysis is the same as that of fresh PPhen-[bipy-$RuCl_4$]H.

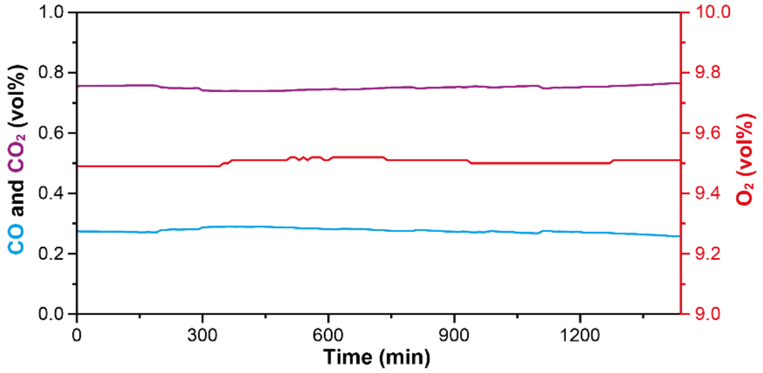

Figure S21 On stream stability test for -[bipy-Ru(II)$(CO)_2Br_2$] at 60,000 mL·g^{-1}·h^{-1} and 453 K.

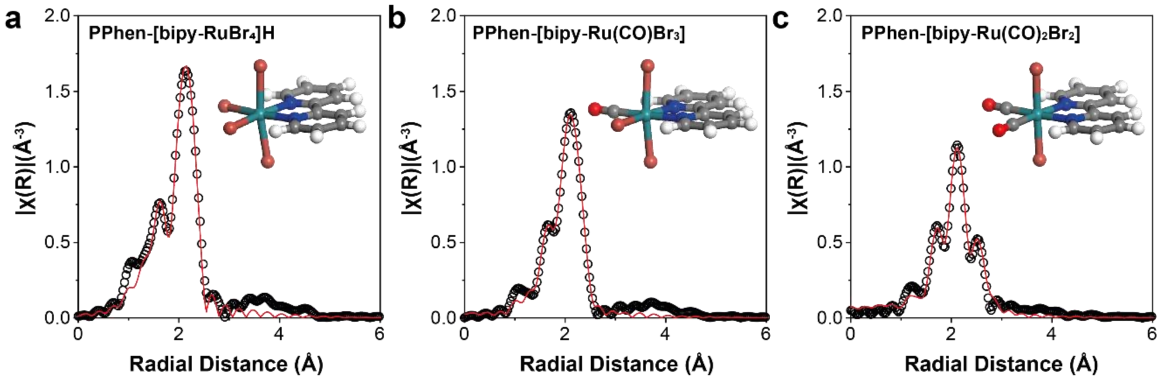

Figure S22. Ru K-edge EXAFS fitting results of (a) -[bipy-Ru(III)Br_4]$^-$, (b) -[bipy-Ru(III)Br_3(CO)] and (c) -[bipy-Ru(II)$Br_2(CO)_2$].

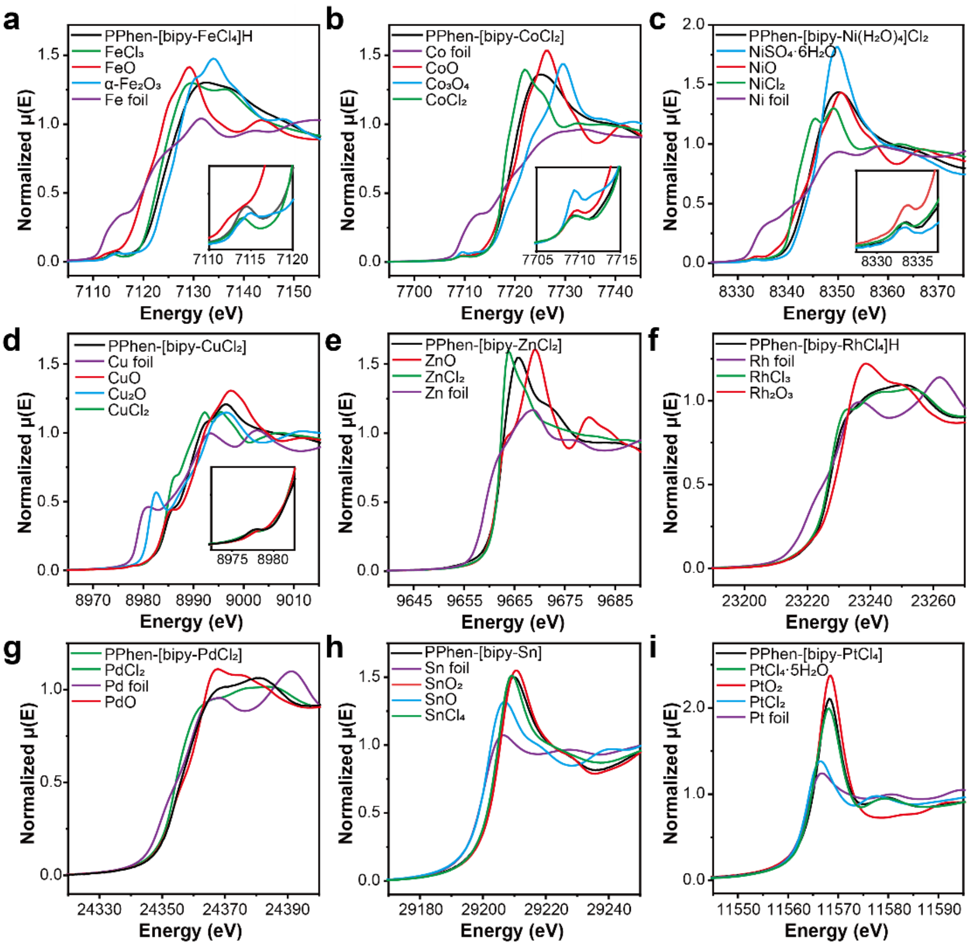

Figure S23. XANES of PPhen-[bipy-MX_n] single sites in comparison with the standard spectrum of metal foils, metal oxides and metal chlorides. (a) PPhen-[bipy-$FeCl_4$]H, (b) PPhen-[bipy-$CoCl_2$], (c) PPhen-[bipy-$Ni(H_2O)_4$]Cl_2, (d) PPhen-[bipy-$CuCl_2$], (e) PPhen-[bipy-$ZnCl_2$], (f) PPhen-[bipy-$RhCl_4$]H, (g) PPhen-[bipy-$PdCl_2$], (h) PPhen-[bipy-Sn], (i) PPhen-[bipy-$PtCl_4$]. Based on the edge shifts, white line intensities and pre-edge features, the oxidation states of these metal cations were identified: Fe(III), Co(II), Ni(II), Cu(II), Zn(II), Rh(III), Pd(II), Sn(IV) and Pt(IV).

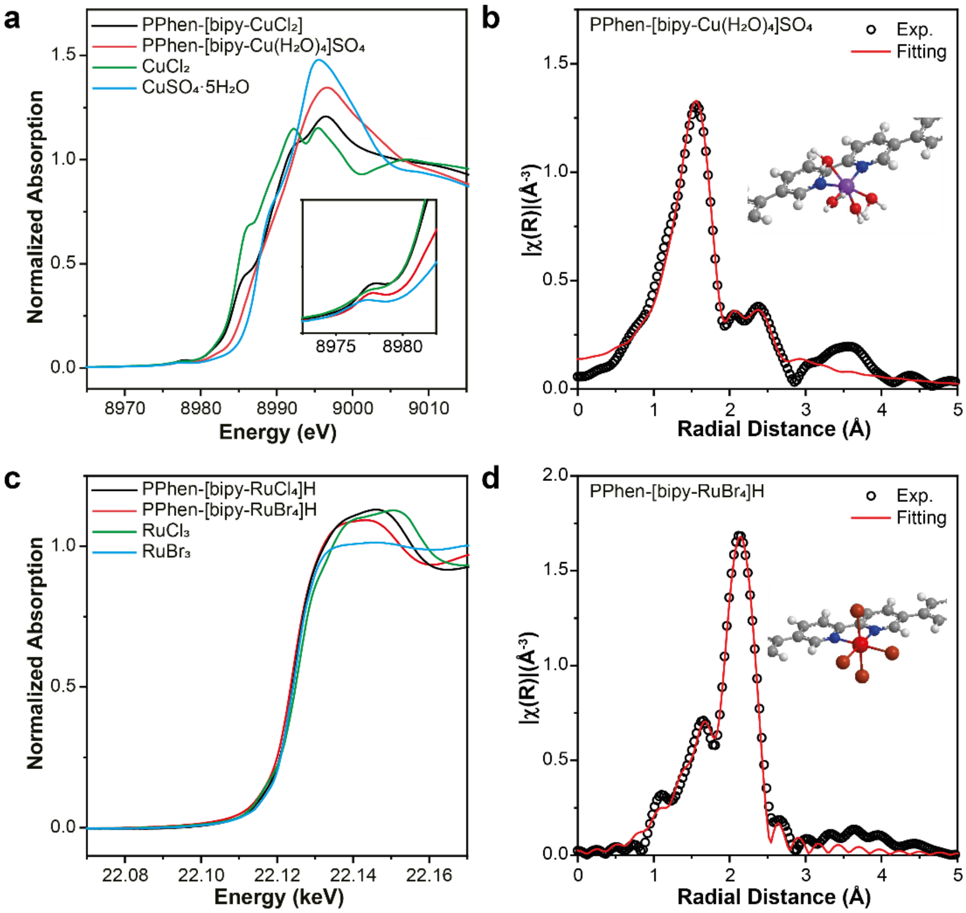

Figure S24. XANES and EXAFS fitting of PPhen-[bipy-Cu(H_2O)$_4$]SO_4 and PPhen-[bipy-$RuBr_4$]H. Cu K-edge (a) XANES and (b) EXAFS fitting of PPhen-[bipy-Cu(H_2O)$_4$]SO_4. Ru K-edge (c) XANES and (d) EXAFS fitting of PPhen-[bipy-$RuBr_4$]H. For PPhen-[bipy-Cu(H_2O)$_4$]SO_4, the pre-edge feature at 8977.7 eV indicates the valence state is Cu(II). Compared with PPhen-[bipy-$CuCl_2$], the two absorption features at 8985.8 eV and 8992.6 eV, which are result from 1*s*→4p_z and 1*s*→4$p_{x,y}$ transitions, become much less significant. Such difference is in consistent with the XANES feature of $CuCl_2$ and $CuSO_4$·5H_2O, suggesting that the coordination geometry of PPhen-[bipy-Cu(H_2O)$_4$]SO_4 and PPhen-[bipy-$CuCl_2$] is Octahedral and Tetrahedral respectively. For PPhen-[bipy-$RuBr_4$]H, the absorption edge position, which is almost identical to $RuBr_3$, proves the valence state is Ru(III). The fitting results are listed in Table S3. The bond length values for both PPhen-[bipy-Cu(H_2O)$_4$]SO_4 and PPhen-[bipy-$RuBr_4$]H are in good agreement with molecular complex listed in Table S3. In sum, the selection of ligands for single sites is also controllable with this PPhen-bipy platform. It is worth mentioning that, the scattering features in the EXAFS between 2-3 Å are mainly due to the scattering from the four N-bonded carbon atoms of the bipy ligand. The other scattering features above 3 Å are attributed to multiple scattering. In comparison with 3*d* metals, the intensity of long path single scattering between 4*d* (or 5*d*) metal centre with these light back scatterers (especially far aromatic carbon atoms) is significantly reduced. This is the reason that the M-C scattering between 2-3 Å and multiple scattering above 3 Å decreased while the atomic number increased.

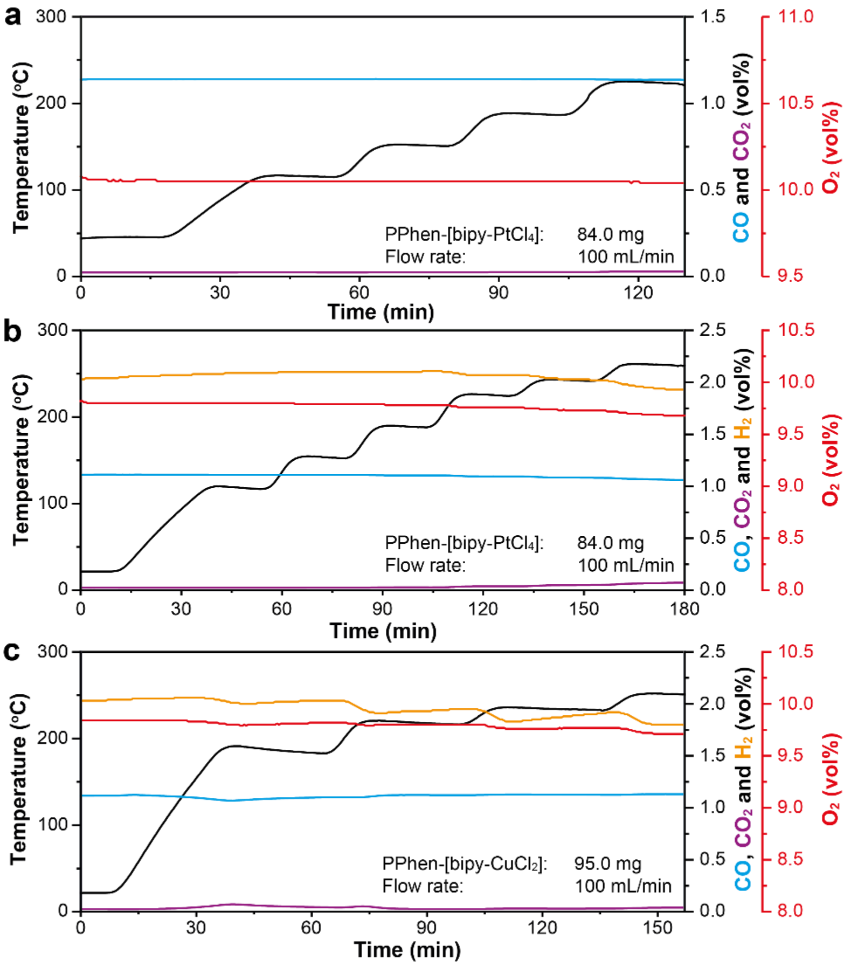

Figure S25. Outlet gas profile and temperature profile of CO oxidation catalysed by (a,b) PPhen-[bipy-$PtCl_4$] and (c) PPhen-[bipy-$CuCl_2$]. Black: temperature, Blue: CO, purple: CO_2, red: O_2, orange: H_2.

Supporting Tables

Table S1. EXAFS fitting results of PPhen-[Ru(bipy)Cl_4]H, PPhen-*mer*(Cl)-[bipy-Ru(CO)Cl_3] and PPhen-*cis*(CO)-*trans*(Cl)-[bipy-Ru$(CO)_2Cl_2$].

Sample	Ru-N [a]		Ru-Cl		Ru-CO	
	C.N.	R	C.N.	R	C.N.	R
-[bipy-RuCl_4]$^-$	2.0	2.05 ± 0.02	4.0 ± 0.4	2.35 ± 0.01	NA	NA
mer(Cl)-[bipy-Ru(CO)Cl_3]	2.0	2.09 ± 0.02	3.2 ± 0.2	2.35 ± 0.01	1.1 ± 0.2	1.88 ± 0.02
cis(CO)-*trans*(Cl)-[bipy-Ru$(CO)_2Cl_2$]	2.0	2.10 ± 0.01	2.2 ± 0.3	2.39 ± 0.01	1.9 ± 0.3	1.88 ± 0.01
cis(CO)-*trans*(Cl)-[bipy-Ru$(CO)_2Cl_2$] after catalysis	2.0	2.09 ± 0.01	1.8 ± 0.3	2.40 ± 0.01	2.1 ± 0.3	1.88 ± 0.01

[a] The coordination number of Ru-N is set to 2.0 as a fixed parameter for EXAFS fitting.

Table S2. Bond length calculated from the structures in Fig. S4 and Fig. S21.

Bond length [Å]	Ru-Cl [a]	Ru-N	Ru-C	C-O	Structure
[Ru(bipy)Cl_4]$^-$	2.412(ax), 2.400(eq)	2.096			S4a
mer(Cl)-[Ru(bipy)(CO)Cl_3]	2.382(ax), 2.353(eq)	2.121, 2.133	1.908	1.139	S4b
cis(CO)-*trans*(Cl)-[Ru(bipy)$(CO)_2Cl_2$]	2.339	2.103	2.002	1.131	S4c
[Ru(bipy)Br_4]$^-$	2.588(ax), 2.483(eq)	2.134			S21a
[Ru(bipy)(CO)Br_3]	2.510(ax), 2.476(eq)	2.148, 2.133	1.899	1.141	S21b
[Ru(bipy)$(CO)_2Br_2$]	2.461	2.117	1.982	1.132	S21c

[a] The (ax) and (eq) are initials for axial position and equatorial position respectively.

Table S3. EXAFS fitting results of PPhen-[bipy-MX_n] and reference materials.

Entry	Name	Coordination Geometry	Valence states	M-N		M-Cl / M-O		M-C / M-CO	
				C.N.	R	C.N.	R	C.N.	R
1	PPhen-[bipy-$FeCl_4$]H	Octahedral	Fe(III)	2.0	1.98±0.06	3.8±0.5	2.25±0.03	4.0	3.01±0.07<br>3.11±0.07
2	PPhen-[bipy-$CoCl_2$]	Tetrahedral	Co(II)	2.0	2.02±0.03	2.1±0.2	2.28±0.02	4.0	2.87±0.06<br>3.02±0.06
3	PPhen-[bipy-$Ni(H_2O)_4$]Cl_2	Octahedral	Ni(II)	2.0	2.07±0.01	4.4±0.7	2.07±0.01	4.0	2.87±0.05<br>3.02±0.05
4	PPhen-[bipy-$Cu(H_2O)_4$]SO_4	Disordered Octahedral	Cu(II)	2.0	1.98±0.07	3.8±0.4	1.99±0.05	4.0	2.82±0.02<br>2.95±0.02
5	PPhen-[bipy-$CuCl_2$]	Disordered Square Planar	Cu(II)	2.0	1.98±0.02	2.0±0.2	2.22±0.01	4.0	2.82±0.02<br>2.95±0.02
6	PPhen-[bipy-$ZnCl_2$]	Tetrahedral	Zn(II)	2.0	1.99±0.05	2.0±0.2	2.22±0.01	4.0	2.86±0.02<br>3.03±0.02
7	PPhen-[bipy-$RuCl_4$]H	Octahedral	Ru(III)	2.0	2.05±0.02	4.0±0.4	2.35±0.01		
8	PPhen-[bipy-$RuBr_4$]H	Octahedral	Ru(III)	2.0	2.07±0.02	4.4±0.4	2.50±0.02		
9	PPhen-[bipy-$RhCl_4$]H	Octahedral	Rh(III)	2.0	2.03±0.02	4.1±0.4	2.34±0.01		
10	PPhen-[bipy-$PdCl_2$]	Square Planar	Pd(II)	2.0	2.04±0.02	2.2±0.3	2.29±0.01		
11	PPhen-[bipy-$PtCl_4$]	Octahedral	Pt(IV)	2.0	2.03±0.02	4.1±0.4	2.30±0.01		
12	Ref[10]: $[Fe(bipy)Cl_4]^-$	Octahedral	Fe(III)	2.0	2.19, 2.20	4.0	2.29, 2.31,<br>2.38, 2.40	4.0	3.06, 3.15
13	Ref[11]:$[Co(bipy)Cl_2]$	Tetrahedral	Co(II)	2.0	2.04, 2.05	2.0	2.22, 2.23	4.0	2.87, 3.04
14	Ref[12]: $[Ni(bipy)(H_2O)_4]$	Octahedral	Ni(II)	2.0	2.06, 2.07	4.0	2.05, 2.07,<br>2.07, 2.08	4.0	2.91, 3.06
15	Ref[13]: $[Cu(bipy)(H_2O)_4]^{2+}$	Disordered Octahedral	Cu(II)	2.0	1.97, 2.00	4.0	1.96, 2.00,<br>2.36, 2.40	4.0	2.81, 2.82,<br>2.98, 3.00
16	Ref[14]:<br>[Cu(*t*-Bu-bipy)Cl_2]	Disordered Square Planar	Cu(II)	2.0	1.97, 1.98	2.0	2.22, 2.22	4.0	2.83, 2.84,<br>2.95, 2.96
17	Ref[15]: $[Zn(bipy)Cl_2]$	Tetrahedral	Zn(II)	2.0	2.06, 2.07	2.0	2.24	4.0	2.89, 3.06
18	Ref[16]: $[Ru(bipy)Cl_4]^-$	Octahedral	Ru(III)	2.0	2.02	4.0	2.36, 2.37		
19	Ref[17]: $[Ru(bipy)(CO)_2Br_2]$	Octahedral	Ru(II)	2.0	2.11, 2.12	2.0	2.52	2.0	1.87, 1.88
20	Ref[18]: $[Rh(bipy)(MeOH)Cl_3]$	Octahedral	Rh(III)	2.0	1.99, 2.00	4.0	2.32, 2.35,<br>2.36		
21	Ref[19]: $[Pd(bipy)Cl_2]$	Square Planar	Pd(II)	2.0	2.01, 2.02	2.0	2.27, 2.29		
22	Ref[20]: $[Pt(bipy)Cl_4]$	Octahedral	Pt(IV)	2.0	2.04	2.0	2.31, 2.32		

Table S4. BET analysis results of PPhen-[bipy-$RuCl_4$]H and PPhen-[bipy-$RhCl_4$]H.

Sample	BET surface area [m^2/g]	Pore volume [cm^3/g]	BET pore size [Å]	BJH pore size [Å]
PPhen-[bipy-$RuCl_4$]H	464.9	0.389	33.5	45.9
PPhen-[bipy-$RhCl_4$]H	410.4	0.270	26.3	49.2

Table S5. Comparison of UV-Vis, IR, and bond length data of Ru complexes in the literatures.

Name	Valence	UV [nm]	IR [cm^{-1}]	Bond length [Å]	Ref
**A**	Ru (III)	400, 365, 294, 254			Ref[21]
**B**	Ru (III)		330 v(Ru-Cl)		Ref[22]
**C**	Ru (III)	372			Ref[23]
**D**	Ru (III)		2064 v(C≡O)		Ref[24]
			2040 v(C≡O)<br>320 v(Ru-Cl)	Ru-C (1.89)<br>Ru-N (2.08, 2.09)<br>Ru-Cl (2.33, 2.34)	Ref[25]
**E**	Ru (II)	354, 313, 300, 284	2055 v(C≡O)<br>1997 v(C≡O)		Ref[26]
			2060 v(C≡O)<br>1995 v(C≡O)<br>327 v(Ru-Cl)		Ref[27]
			2065 v(C≡O)<br>2004 v(C≡O)		Ref[28]
			2060 v(C≡O)<br>2000 v(C≡O)		Ref[29]
		363			Ref[30]
			2057 v(C≡O)<br>1998 v(C≡O)		Ref[31]
		352	2066 v(C≡O)<br>2004 v(C≡O)		Ref[32]
		354, 313, 300, 284	2055 v(C≡O)<br>1997 v(C≡O)<br>330 v(Ru-Cl)		Ref[33]
			2030 v(C≡O)<br>1980 v(C≡O)<br>330 v(Ru-Cl)		Ref[34]
			2066 v(C≡O)<br>2003 v(C≡O)	Ru-Cl (2.39, 2.39)<br>Ru-N (2.11, 2.10)<br>Ru-C (1.84, 1.82)	Ref[35]
		352	2067 v(C≡O)<br>2001 v(C≡O)		Ref[36]
		352, 313, 300, 286, 246	2059 v(C≡O)<br>1999 v(C≡O)<br>338 v(Ru-Cl)		Ref[37]
			2065 v(C≡O)<br>2004 v(C≡O)	Ru-Cl (2.37, 2.38)<br>Ru-N (2.12, 2.11)<br>Ru-C (1.96, 1.90)	Ref[38]
**F**	Ru (II)	500, 448, 360, 311, 300	2040 v(C≡O)<br>1980 v(C≡O)<br>330 v(Ru-Cl)<br>302 v(Ru-Cl)		Ref[33]
		red brown	2066 v(C≡O)<br>2055 v(C≡O)		Ref[39]
			2040 v(C≡O)<br>1980 v(C≡O)	Ru-Cl (2.44, 2.41)<br>Ru-N (2.12, 2.09)<br>Ru-C (1.90, 1.94)	Ref[35]

Name	Valence	UV [nm]	IR [cm^{-1}]	Bond length [Å]	Ref
				Ru-Cl (2.42, 2.40)<br>Ru-N (2.10, 2.07)<br>Ru-C (1.87, 1.86)	Ref[38]
		352	2067 *v*(C≡O)<br>2001 *v*(C≡O)		Ref[36]
		303, 293, 248, 222	2051 *v*(C≡O)<br>1986 *v*(C≡O)<br>319 *v*(Ru-Cl)<br>297 *v*(Ru-Cl)		Ref[37]
**G**	Ru (II)			DFT:<br>Ru-Cl (2.44, 2.44)<br>Ru-N (2.10, 2.10)<br>Ru-C (1.96, 1.96)	Ref[38]
**H**	Ru (II)	410, 295, 252	2067 *v*(C≡O)<br>2059 *v*(C≡O)<br>1998 *v*(C≡O)		Ref[40]
			2064 *v*(C≡O)<br>2001 *v*(C≡O)		Ref[41]
		352, 300, 286, 246	2055 *v*(C≡O)<br>1995 *v*(C≡O)		Ref[42]
		354	2055 *v*(C≡O)<br>1995 *v*(C≡O)		Ref[23]
**I**	Ru (II)		2035 *v*(C≡O)<br>1966 *v*(C≡O)	Ru-Cl (2.52)<br>Ru-N (2.12, 2.12)<br>Ru-C (1.87, 1.86)	Ref[35]
			2068 *v*(C≡O)<br>2005 *v*(C≡O)	Ru-Cl (2.52)<br>Ru-N (2.12, 2.12)<br>Ru-C (1.86, 1.85)	Ref[38]
**J**	Ru (II)		2140 *v*(C≡O)<br>2086 *v*(C≡O)<br>2068 *v*(C≡O)	Ru-Cl (2.38)<br>Ru-N (2.10, 2.10)<br>Ru-C (1.94, 1.93, 1.92)	Ref[38]

Compound:

A. H(bpy)[RuCl$_4$(bipy)]·H$_2$O
B. [Ru(bipy)Cl$_3$]
C. [Ru(bipy)(CO)Cl$_3$]
D. *mer(Cl)*-[Ru(bipy)(CO)Cl$_3$]
E. *cis*(CO)-*trans*(Cl)-[Ru(bipy)(CO)$_2$Cl$_2$]
F. *cis*(CO)-*cis*(Cl)-[Ru(bipy)(CO)$_2$Cl$_2$]
G. *trans*(CO)-*cis*(Cl)-[Ru(bipy)(CO)$_2$Cl$_2$]
H. [Ru(bipy)(CO)$_2$Cl$_2$]
I. [Ru(bipy)(CO)$_2$ClH]
J. *fac*-[Ru(bipy)(CO)$_3$Cl]Cl

Table S6. Peak fitting results of Cl K-edge, Ru L_3-edge and Ru L_2-edge XANES of PPhen-[bipy-Ru(III)Cl_4]H and PPhen-*cis*(CO)-*trans*(Cl)-[bipy-Ru(II)$(CO)_2Cl_2$].

Sample	Edge	Peak	Electron Transition	Energy [eV]	Δ_0 [eV] [a]
**PPhen-[bipy-Ru(III)Cl_4]H**	Cl K-edge	***D***	Cl 1*s* → Ru 4*d* t_{2g}	2820.8	3.0
		E	Cl 1*s* → Ru 4*d* e_g	2823.8	
	Ru L_3-edge	***A***	Ru $2p_{3/2}$ → Ru 4*d* t_{2g}	2838.0	2.9
		B	Ru $2p_{3/2}$ → Ru 4*d* e_g	2840.9	
	Ru L_2-edge	***F***	Ru $2p_{1/2}$ → Ru 4*d* t_{2g}	2966.8	2.8
		G	Ru $2p_{1/2}$ → Ru 4*d* e_g	2969.6	
**PPhen-*cis*(CO)-*trans*(Cl)-[bipy-Ru(II)$(CO)_2Cl_2$]**	Cl K-edge	***E***	Cl 1*s* → Ru 4*d* e_g	2823.7	N/A
	Ru L_3-edge	***B***	Ru $2p_{3/2}$ → Ru 4*d* e_g	2841.0	
		C	Ru $2p_{3/2}$ → CO 2*p* π*	2843.0	
	Ru L_2-edge	***G***	Ru $2p_{1/2}$ → Ru 4*d* e_g	2969.7	
		H	Ru $2p_{1/2}$ → CO 2*p* π*	2972.0	

[a] This Ligand filed splitting energy Δ_0 was measured with core hole states, so the value obtained by this method would be slightly different from other methods (UV-Vis, DFT).

Table S7. Ru K-edge positions of PPhen-[bipy-Ru(III)Cl_4]H, PPhen-*cis*(CO)-*trans*(Cl)-[bipy-Ru(II)$(CO)_2Cl_2$] and reference standard materials.

Compound	Valence	Edge position (1) [a]	Edge position (2) [b]
Ru foil	Ru(0)	22117.0 eV	22120.9 eV
$RuCl_3$	Ru(III)	22125.8 eV	22124.5 eV
RuO_2	Ru(IV)	22132.5 eV	22127.2 eV
$[Ru(NH_3)_6]Cl_2$	Ru(II)	22126.4 eV	22123.2 eV
$[Ru(NH_3)_6]Cl_3$	Ru(III)	22128.2 eV	22125.1 eV
$[Ru(NH_3)_5Cl]Cl_2$	Ru(III)	22127.5 eV	22124.6 eV
PPhen-[bipy-RuCl4]H	Ru(III)	22124.4 eV	22123.7 eV
PPhen-cis(CO)-trans(Cl)-[bipy-Ru(CO)2Cl2]	Ru(II)	22125.8 eV	22124.4 eV

[a] This edge position is determined by the peak position of highest absorption feature in the first derivative XANES. [b] This edge position is taken from the photon energy value at the 0.5 edge jump of normalized XANES.

Table S8. XPS binding energy of PPhen-[bipy-Ru(III)Cl_4]H and PPhen-*cis*(CO)-*trans*(Cl)-[bipy-Ru(II)$(CO)_2Cl_2$] at C 1*s* and Ru 3*d* region.

Sample	Ru $3d_{5/2}$	Ru $3d_{3/2}$	C 1*s* (C-C/C-H)	C 1*s* (C-N)
PPhen-[bipy-Ru(III)Cl_4]H	282.4 eV	286.6 eV	284.8 eV	286.2 eV
PPhen-*cis*(CO)-*trans*(Cl)-[bipy-Ru(II)$(CO)_2Cl_2$]	281.8 eV	286.0 eV	284.8 eV	286.4 eV

Table S9. Comparison of TOF and E_a of -[bipy-Ru(II)(CO)$_2$Cl$_2$] with the reported values for other catalysts in the literatures.

Ref	Active Sites	CO	O_2	CO Conversion	WHSV [a] (mL·g_{cat}^{-1}·h^{-1})	TOF (×10^{-2}·s^{-1})	Temperature (K)	E_a (kJ·mol^{-1})
[43]	4 nm Ru NPs					1,000	513	113.4
[43]	2 nm Ru NPs					300	513	130.2
[44]	5 wt% Ru/SiO_2	9.8 Torr	4.9 Torr			163	450	94.5
[45]	3 wt% Ru/SiO_2	32 Torr	8 Torr			0.6	393	93.24
[45]	3 wt% Ru/SiO_2	26.7 Torr	13.3 Torr			1	393	106.26
[45]	3 wt% Ru/SiO_2	13.3 Torr	26.7 Torr			0.4	393	87.36
[46]	3 wt % Ru/MgO	13.7 Torr	6.8 Torr					82.32
[47]	Ru (0001)	16 Torr	8 Torr					81.9
[48]	Pt(110)	8 Torr	4 Torr			50	500	100.8
[48]	Pd(111)	8 Torr	4 Torr			180	500	100.8
[48]	RuO_2(110)	8 Torr	4 Torr			1060	500	100.8
[48]	Ru(0001)	8 Torr	4 Torr			1800	500	79.8
[49]	2 wt% Ru supported on $[Ca_{24}Al_{28}O_{64}]^{4+}(e^-)_4$	9.52%	4.72%		25200	19	393	58.8
[49]	2 wt% Ru/TiO_2	9.52%	4.72%			6	393	79.8
[49]	2 wt% Ru/Al_2O_3	9.52%	4.72%			2.5	393	130.2
[49]	2 wt% Ru supported on $[Ca_{24}Al_{28}O_{64}]^{4+}(O^{2-})_2$	9.52%	4.72%			1.5	393	79.8
[50]	Non-oxidic RuO_2(110) layer	20 mbar	10 mbar			400	500	75.6
[50]	Thick oxide RuO_2(110) layer	20 mbar	10 mbar			300	500	75.6
[51]	RuO_2(110)	1400 Pa	550Pa			400	488	78.12
[52]	3% Ru/SiO_2	10%	10%		18000	0.1	333	81.9
[52]	3% Ru/SiO_2	10%	10%		18000	0.4	333	81.9
[52]	3% Ru/MgO	10%	10%		18000	0.05	315	81.9
[53]	Ru/RuO_2	1.8%	0.9%	35%	240,000	19.9	453	86
[54]	Isolated Pt sites on CeO_2	0.4%	10%	12.6%	200,000	2.43	373	43
[55]	Pt_{iso}/TiO_2 reduced at 523 K	1%	1%	0.03%	480,000	0.14	423	78
[55]	Pt_{iso}/TiO_2 reduced at 723 K			0.11%		0.49	423	48
[56]	Pt_1/CeO_2-a	0.1%	5%	0.8%	2,400,000	1.7	423	86
[56]	Pt-O-Pt/CeO_2-a			91.9%		197	423	40
[57]	Atomic Cu sites on CeO_2	1%	10%	20.9%	120,000	2.48	373	62
[58]	Au NPs on CeO_2	1%	20%	100%	30,000	98.8	513	49
*	[-bipy-Ru(II)(CO)$_2$Cl$_2$] (This work)	1%	10%	2.9%	600,000	0.73	462	90
				6.8%	600,000	1.77	479	
				15%	600,000	3.89	498	

[a] WHSV = Weight hourly space velocity per gram of catalysts.

References

[1] a) A. J. Dent, G. Cibin, S. Ramos, S. A. Parry, D. Gianolio, A. D. Smith, S. M. Scott, L. Varandas, S. Patel, M. R. Pearson, L. Hudson, N. A. Krumpa, A. S. Marsch, P. E. Robbins, *J. Phys. Conf. Ser.* **2013**, *430*; b) A. J. Dent, G. Cibin, S. Ramos, A. D. Smith, S. M. Scott, L. Varandas, M. R. Pearson, N. A. Krumpa, C. P. Jones, P. E. Robbins, *J. Phys. Conf. Ser.* **2009**, *190*.

[2] B. Ravel, M. Newville, *J. Synchrotron Radiat.* **2005**, *12*, 537-541.

[3] M. Frisch, G. W. Trucks, H. B. Schlegel, G. E. Scuseria, M. A. Robb, J. R. Cheeseman, G. Scalmani, V. Barone, B. Mennucci, G. e. Petersson, **2014**.

[4] a) A. D. Becke, *J. Chem. Phys.* **1997**, *107*, 8554-8560; b) C. Lee, W. Yang, R. G. Parr, *Phys. Rev. B* **1988**, *37*, 785-789; c) S. H. Vosko, L. Wilk, M. Nusair, *Can. J. Phys.* **1980**, *58*, 1200-1211; d) P. J. Stephens, F. J. Devlin, C. F. Chabalowski, M. J. Frisch, *J. Phys. Chem.* **1994**, *98*, 11623-11627.

[5] a) W. J. Wolfgong, in *Handbook of Materials Failure Analysis with Case Studies from the Aerospace and Automotive Industries* (Eds.: A. S. H. Makhlouf, M. Aliofkhazraei), Butterworth-Heinemann, Boston, **2016**, pp. 279-307; b) S. Nasrazadani, S. Hassani, in *Handbook of Materials Failure Analysis with Case Studies from the Oil and Gas Industry* (Eds.: A. S. H. Makhlouf, M. Aliofkhazraei), Butterworth-Heinemann, **2016**, pp. 39-54; c) O. D. Neikov, N. A. Yefimov, in *Handbook of Non-Ferrous Metal Powders (Second Edition)* (Eds.: O. D. Neikov, S. S. Naboychenko, N. A. Yefimov), Elsevier, Oxford, **2019**, pp. 3-62; d) D. E. Newbury, N. W. M. Ritchie, *Microsc. Microanal.* **2016**, *22*, 520-535; e) D. E. Newbury*, N. W. M. Ritchie, *Scanning* **2013**, *35*, 141-168; f) P. Kuisma-Kursula, *X-Ray Spectrom.* **2000**, *29*, 111-118.

[6] Y. Leng, *Materials characterization: introduction to microscopic and spectroscopic methods*, John Wiley & Sons, **2009**.

[7] B. E. Van Kuiken, M. Valiev, S. L. Daifuku, C. Bannan, M. L. Strader, H. N. Cho, N. Huse, R. W. Schoenlein, N. Govind, M. Khalil, *J. Phys. Chem. A* **2013**, *117*, 4444-4454.

[8] B. E. Van Kuiken, N. Huse, H. Cho, M. L. Strader, M. S. Lynch, R. W. Schoenlein, M. Khalil, *J. Phys. Chem. Lett.* **2012**, *3*, 1695-1700.

[9] a) A. A. Hummer, P. Heffeter, W. Berger, M. Filipits, D. Batchelor, G. E. Buchel, M. A. Jakupec, B. K. Keppler, A. Rompel, *J. Med. Chem.* **2013**, *56*, 1182-1196; b) L. Salassa, T. Ruiu, C. Garino, A. M. Pizarro, F. Bardelli, D. Gianolio, A. Westendorf, P. J. Bednarski, C. Lamberti, R. Gobetto, P. J. Sadler, *Organometallics* **2010**, *29*, 6703-6710.

[10] V. Amani, N. Safari, H. R. Khavasi, *Polyhedron* **2007**, *26*, 4257-4262.

[11] T. Kojima, D. Kumaki, J.-i. Nishida, S. Tokito, Y. Yamashita, *J. Mater. Chem.* **2011**, *21*, 6607-6613.

[12] S. Boonlue, C. Theppitak, K. Chainok, *Acta Crystallogr. E* **2012**, *68*, m908.

[13] C. Yamamoto, H. Nishikawa, M. Nihei, T. Shiga, M. Hedo, Y. Uwatoko, H. Sawa, H. Kitagawa, Y. Taguchi, Y. Iwasa, H. Oshio, *Inorg. Chem.* **2006**, *45*, 10270-10276.

[14] D. J. Awad, U. Schilde, P. Strauch, *Inorg. Chim. Acta* **2011**, *365*, 127-132.

[15] E. Nauha, P. Naumov, M. Lusi, *Crystengcomm* **2016**, *18*, 4699-4703.

[16] B. Durham, D. I. Cox, A. W. Cordes, S. Barsoum, *Acta Crystallographica Section C* **1990**, *46*, 321-322.

[17] M. Haukka, M. Ahlgrén, T. A. Pakkanen, *J. Chem. Soc., Dalton Trans.* **1996**, 1927-1933.

[18] R. Bieda, I. Ott, M. Dobroschke, A. Prokop, R. Gust, W. S. Sheldrick, *J. Inorg. Biochem.* **2009**, *103*, 698-708.

[19] R. A. Gutierrez Marquez, C. Crisostomo-Lucas, D. Morales-Morales, S. Hernandez-Ortega, *Acta Crystallogr. E* **2014**, *70*, m218.

[20] N. Marino, C. H. Fazen, J. D. Blakemore, C. D. Incarvito, N. Hazari, R. P. Doyle, *Inorg. Chem.* **2011**, *50*, 2507-2520.

[21] T. Togano, N. Nagao, M. Tsuchida, H. Kumakura, K. Hisamatsu, F. S. Howell, M. Mukaida, *Inorg. Chim. Acta* **1992**, *195*, 221-225.

[22] N. C. Pramanik, S. Bhattacharya, *Polyhedron* **1997**, *16*, 3047-3053.

[23] M. N. Collombdunandsauthier, A. Deronzier, R. Ziessel, *J. Electroanal. Chem.* **1991**, *319*, 347-353.

[24] F. Hartl, A. K. Renfrew, F. Lafolet, T. Mahabiersing, M. J. Calhorda, S. Chardon-Noblat, M. Haukka, A. Deronzier, *Inorg. Chem.* **2009**, *48*, 8233-8244.

[25] F. P. Pruchnik, E. Galdecka, Z. Galdecki, A. Kowalski, *Polyhedron* **1999**, *18*, 2091-2097.

[26] G. B. Deacon, J. M. Patrick, B. W. Skelton, N. C. Thomas, A. H. White, *Aust. J. Chem.* **1984**, *37*, 929-945.

[27] D. S. Black, G. B. Deacon, N. C. Thomas, *Aust. J. Chem.* **1982**, *35*, 2445-2453.

[28] M. Lutz, B. Findeis, M. Haukka, T. A. Pakkanen, L. H. Gade, *Eur. J. Inorg. Chem.* **2001**, 3155-3162.

[29] E. Eskelinen, T. J. J. Kinnunen, M. Haukka, T. A. Pakkanen, *Eur. J. Inorg. Chem.* **2002**, 1169-1173.

[30] G. Balducci, E. Iengo, N. Demitri, E. Alessio, *Eur. J. Inorg. Chem.* **2015**, 4296-4311.

[31] P. A. Anderson, G. B. Deacon, K. H. Haarmann, F. R. Keene, T. J. Meyer, D. A. Reitsma, B. W. Skelton, G. F. Strouse, N. C. Thomas, J. A. Treadway, A. H. White, *Inorg. Chem.* **1995**, *34*, 6145-6157.

[32] A. Gabrielsson, S. Zalis, P. Matousek, M. Towrie, A. Vlcek, *Inorg. Chem.* **2004**, *43*, 7380-7388.

[33] J. M. Kelly, C. M. Oconnell, J. G. Vos, *Inorg. Chim. a-Lett* **1982**, *64*, L75-L76.

[34] K. Joseph, S. S. Deshpande, S. A. Pardhy, I. R. Unny, S. K. Pandit, S. Gopinathan, C. Gopinathan, *Inorg Chim a-Art Let* **1984**, *82*, 59-61.

[35] M. Haukka, J. Kiviaho, M. Ahlgren, T. A. Pakkanen, *Organometallics* **1995**, *14*, 825-833.

[36] E. Eskelinen, M. Haukka, T. Venalainen, T. A. Pakkanen, M. Wasberg, S. Chardon-Noblat, A. Deronzier, *Organometallics* **2000**, *19*, 163-169.

[37] S. ChardonNoblat, A. Deronzier, R. Ziessel, D. Zsoldos, *Inorg. Chem.* **1997**, *36*, 5384-5389.

[38] M. Haukka, P. Hirva, S. Luukkanen, M. Kallinen, M. Ahlgren, T. A. Pakkanen, *Inorg. Chem.* **1999**, *38*, 3182-3189.

[39] J. V. Kingston, J. W. Jamieson, Wilkinso.G, *J. Inorg. Nucl. Chem.* **1967**, *29*, 133-&.

[40] N. Ishito, H. Kobayashi, K. Nakajima, Y. Maegawa, S. Inagaki, K. Hara, A. Fukuoka, *Chem.-Eur. J.* **2015**, *21*, 15564-15569.

[41] D. Mulhern, S. Brooker, H. Gorls, S. Rau, J. G. Vos, *Dalton Trans.* **2006**, 51-57.

[42] M. N. Collombdunandsauthier, A. Deronzier, R. Ziessel, *J. Organomet. Chem.* **1993**, *444*, 191-198.

[43] S. H. Joo, J. Y. Park, J. R. Renzas, D. R. Butcher, W. Huang, G. A. Somorjai, *Nano Lett.* **2010**, *10*, 2709-2713.

[44] N. W. Cant, P. C. Hicks, B. S. Lennon, *J. Catal.* **1978**, *54*, 372-383.

[45] J. T. Kiss, R. D. Gonzalez, *J. Phys. Chem.* **1984**, *88*, 892-897.

[46] J. Assmann, V. Narkhede, L. Khodeir, E. Löffler, O. Hinrichsen, A. Birkner, H. Over, M. Muhler, *J. Phys. Chem. B* **2004**, *108*, 14634-14642.

[47] C. H. Peden, D. W. Goodman, *J. Phys. Chem.* **1986**, *90*, 1360-1365.

[48] F. Gao, D. W. Goodman, *Phys. Chem. Chem. Phys.* **2012**, *14*, 6688-6697.

[49] M. J. Sharif, M. Kitano, Y. Inoue, Y. Niwa, H. Abe, T. Yokoyama, M. Hara, H. Hosono, *J. Phys. Chem. C* **2015**, *119*, 11725-11731.

[50] H. Over, O. Balmes, E. Lundgren, *Surf. Sci.* **2009**, *603*, 298-303.

[51] H. Over, O. Balmes, E. Lundgren, *Catal. Today* **2009**, *145*, 236-242.

[52] J. Aßmann, E. Löffler, A. Birkner, M. Muhler, *Catal. Today* **2003**, *85*, 235-249.

[53] N. Vijay, A. Jens, M. Martin, *Zeitschrift für Physikalische Chemie* **2005**, *219*, 979-995.

[54] L. Nie, D. Mei, H. Xiong, B. Peng, Z. Ren, X. I. P. Hernandez, A. DeLaRiva, M. Wang, M. H. Engelhard, L. Kovarik, A. K. Datye, Y. Wang, *Science* **2017**, *358*, 1419.

[55] L. DeRita, J. Resasco, S. Dai, A. Boubnov, H. V. Thang, A. S. Hoffman, I. Ro, G. W. Graham, S. R. Bare, G. Pacchioni, X. Pan, P. Christopher, *Nat. Mater.* **2019**, *18*, 746-751.

[56] H. Wang, J.-X. Liu, L. F. Allard, S. Lee, J. Liu, H. Li, J. Wang, J. Wang, S. H. Oh, W. Li, M. Flytzani-Stephanopoulos, M. Shen, B. R. Goldsmith, M. Yang, *Nat. Commun.* **2019**, *10*, 3808.

[57] L. Kang, B. Wang, Q. Bing, M. Zalibera, R. Büchel, R. Xu, Q. Wang, Y. Liu, D. Gianolio, C. C. Tang, E. K. Gibson, M. Danaie, C. Allen, K. Wu, S. Marlow, L.-d. Sun, Q. He, S. Guan, A. Savitsky, J. J. Velasco-Vélez, J. Callison, C. W. M. Kay, S. E. Pratsinis, W. Lubitz, J.-y. Liu, F. R. Wang, *Nat. Commun.* **2020**, *11*, 4008.

[58] J. Wang, H. Tan, S. Yu, K. Zhou, *ACS Catal.* **2015**, *5*, 2873-2881.

Author Contributions

L.K. and F.R.W. conceived the study. L.K. and B.W. carried out materials synthesis. L.K. performed materials characterization and catalytic evaluations. L.K., B.W., E.K.G. and F.R.W. performed *ex situ* and *in situ* XAFS study. H.A. performed the Ru L-edge and Cl K-edge XAFS study. L.K., M.D. and Q.H. conducted the STEM measurement. K.W. and L.D.S. performed far-IR analysis. A.T. and R.C. performed DFT calculation. L.K. and F.R.W. wrote the manuscript and all authors revised the manuscript.
